# Effects of laser irradiation on phytochemical composition, histological anatomy, genetic diversity, and food safety of *Ocimum basilicum* L.

**DOI:** 10.1186/s12870-026-08136-2

**Published:** 2026-02-09

**Authors:** S. F. Desoukey, Hend S. M. Abdel-Aziz, Shaimaa S. Shoman, Amal F. Al-Shafeay, Randa S. Hasan, Essam M. Abdelsalam, Shaimaa R. Ali

**Affiliations:** 1 Department of Agriculture Botany, Faculty of Agriculture, Cairo University, Giza, 12613 Egypt; 2 Department of Biochemistry, Faculty of Agriculture, Cairo University, Giza, 12613 Egypt; 3 Agriculture Genetic Engineering Research Institute (AGERI), Agriculture Research Center (ARC), Giza, 12619 Egypt; 4 Regional Center for Food and Feed (RCFF), Agricultural Research Center (ARC), Giza, 12619 Egypt; 5 Department of Laser Applications in Metrology, Photochemistry and Agriculture (LAMPA), National Institute of Laser Enhanced Sciences (NILES), Cairo University, Giza, 12613 Egypt

**Keywords:** Laser irradiation, *Ocimum basilicum* L., Phytochemicals, Phytohormones, Essential oils, Molecular genetics

## Abstract

Enhancing crop productivity and sustainability remains a major challenge due to the environmental impact of conventional chemical methods. Laser seed bio-stimulation offers an eco-friendly approach to improve plant growth, quality, and stress tolerance. Laser irradiation as a seed pre-treatment offers a sustainable, non-chemical approach that may contribute to improved quality and safety of medicinal and aromatic plants. This study evaluated the effects of red (650 nm) and blue (450 nm) laser irradiation for 5 and 10 min on sweet basil (*Ocimum basilicum* L.) seeds by analyzing vegetative growth, biochemical composition, phytohormones, and genetic variation compared to the control. Results demonstrated that 10-min laser treatment significantly enhanced plant growth, including parameters such as plant height, main stem length, number of leaves per plant, fresh and dry shoot weight, and root length, relative to the control. Laser exposure also strengthened basil leaf anatomy by thickening conducting tissues and increasing total phenolic content, antioxidant activity, pigment concentrations, phytohormone levels (GA₃ and IAA), essential oil yield, and antioxidant enzyme activity. Laser treatment modulated the essential oil composition, with variable responses among individual constituents. The impact of laser irradiation on *Ocimum basilicum* to induce genetic variability and enhance its physiological performance. Basil seeds were exposed to different laser type and wave length, and the resulting plants were evaluated for morphological changes and genomic polymorphism using SCoT and ISSR markers. SCoT and ISSR markers revealed that 10-min laser irradiation induced the most distinct genetic variations. SCoT primers produced 6 polymorphic bands (60%, 290–1300 bp), while ISSR primers detected 82 bands, with 22% polymorphism (150–1700 bp). Cluster analysis showed that 10-min treatments had the lowest genetic similarity, whereas 5-min treatments were more similar to the control. Combining data from both markers confirmed that red and blue laser exposure for 10 min resulted in the lowest similarity scores, while 5-min treatments exhibited the highest similarity. The present findings validate that laser irradiation can act as an eco-friendly bio-stimulation tool to enhance basil growth and biochemical composition. These results highlight its potential applications in sustainable agriculture, though further validation under open-field conditions is still required.

**Supplementary Information:**

The online version contains supplementary material available at 10.1186/s12870-026-08136-2.

## Key Message

“Laser irradiation significantly enhanced basil morphology, phytochemical profile, some essential oil composition, and genetic expression, providing novel insights for improving *Ocimum basilicum *growth and bioactive compounds.”

**Supplementary Information:**

The online version contains supplementary material available at 10.1186/s12870-026-08136-2.

## Introduction

The *Lamiaceae* (or Labiatae) family includes the well-known annual herb sweet basil (*Ocimum basilicum* L.). Native to East Africa and India, basil is a tropical plant. Basil leaves and blossom tips are steam-distilled to extract essential oils, which are then utilized in traditional remedies, dental and oral goods, food flavoring, and fragrances [1]. Additionally, physiologically active components with insecticidal, antibacterial, and antifungal capabilities have been demonstrated to be present in essential oils. Predominant elements of essential oils, including methyl chavicol, eugenol, linalool, camphor, and methyl cinnamate, are responsible for these qualities [2].

The herb *O. basilicum* L. (basil) is widely cultivated as a leafy vegetable, spice and medicinal plant, and is of considerable interest due to its rich profile of secondary metabolites including phenolic acids, flavonoids and essential oils. For example, recent work demonstrated that foliar application of a silicon-rich horsetail extracts significantly enhanced basil’s growth, total phenolic and flavonoid content, antioxidant capacity, and essential oil yield (increases up to ~ 135% compared with control). These bioactive metabolites underpin basil’s use in food, pharmaceutical, cosmetic and agro-industrial applications, and highlight the potential of metabolic-enhancement strategies for value-added production [3].

Sweet basil (*Ocimum basilicum* L.), a member of the Lamiaceae family, is one of the most economically valuable aromatic and medicinal plants due to its rich content of essential oils and secondary metabolites such as linalool, eugenol, and methyl chavicol. These compounds are responsible for the plant’s characteristic aroma as well as its antioxidant, antimicrobial, and therapeutic properties, making basil a key species for the food, pharmaceutical, and cosmetic industries [4].

Previous studies on Lamiaceae species—including *Rosmarinus officinalis*, *Mentha piperita*, and *Thymus vulgaris*—have demonstrated that light quality and other physical stimulation techniques can influence the biosynthesis of essential oils and phenolic compounds. Similarly, recent research on *O. basilicum* has shown that controlled exposure to physical factors, including laser and LED light treatments, can enhance germination, growth, and the accumulation of phytochemicals, thereby supporting its potential role in sustainable and eco-friendly agricultural production systems [5, 6].

Excessive use of chemicals (mineral fertilizers, inhibitors, etc.) to increase plant quality and production results in altered soil structure, contamination of the environment, degradation of living things, and lower-quality products. Therefore, the development and application of contemporary alternative techniques of quality assurance are necessary to ensure the safety of fresh products in the economy. In place of soil additives and fertilizers, physical factors like microwave, laser radiation, magnetic fields, and ultrasonic irradiation can affect plants, or more specifically, their seeds, tubers, bulbs, sprouts, or adult plants at various stages of plant development. These factors can also lower the amount of toxins in raw materials, eco-friendly ways to boost yields and improve food safety [7].

Laser irradiation is one of the most widely used physical techniques for stimulating plant growth. It is also regarded as the most appropriate solution in agricultural physics to boost natural production and has drawn a lot of attention globally for improving plant growth and quality. In agriculture, laser irradiation treatment of seeds and plants is regarded as a novel method. Plants and seeds exposed to various forms of laser irradiation demonstrated favorable outcomes in terms of germination and growth [8]. Recent research indicates that using laser radiation to stimulate seeds can increase germination and growth energy by 20%, shorten plant development phases, create more robust plants, and ultimately increase yield by 11–12%.

Continuous or pulsed laser irradiation of agricultural and medicinal crop seeds and their shoots using a laser beam with a varies wavelength between (632 & 670 nm) and for a predetermined amount of time is known as laser irradiation. The energy from the incident light is converted to chemical energy and causes the seed to undergo physiological and biochemical reactions that may improve the dynamics of its growth [9]. The bio stimulation process depends critically on a seed's capacity to absorb and store radiant energy. The major goal of this strategy is to raise grain yields, minimize morbidity, increase the amount of high-quality green mass and establish a powerful root system of plants. Research has indicated that when seeds are exposed to tiny amounts of laser radiation, plants grow more quickly and produce more fruit [10].

Fresh and minimally processed herbs are highly susceptible to microbial contamination during cultivation, harvesting, and post-harvest handling, posing potential food safety concerns [11]. Laser and light-based irradiation treatments are considered promising non-chemical approaches for enhancing food safety of medicinal and aromatic plants through microbial load reduction while preserving product quality [12].

The plants can react in various approaches in the case of laser exposure as a stress factor in which the plants activate the response to the stimulus by activation of a secondary metabolites synthesis pathways and enhancing production of different types of bioactive compounds as a defense mechanism [13]. Numerous seeds, including wheat, peas, sunflower, soybean, pepper, white lupine, faba bean, tomato, and alfalfa have already shown the benefit of laser bio stimulation in improving their growth characteristics and fruit quality [14–16]. Furthermore, laser treatment has been demonstrated to improve plant resistance to biotic and abiotic stressors [17], and stimulated the activities of antioxidant enzymes which protect cells against oxidative stress [18]. Depending on the source, wavelength, irradiation duration, and irradiation dose, this bio stimulation process can have several beneficial effects, including an increase in the plant's shoot and root length, stalk thickness, number of leaves, dry mass, and levels of chlorophyll a, chlorophyll b, anthocyanins, photosynthesis intensity, and total carbohydrates and carotenoid content [19]. Furthermore, applying red and blue light to seeds can also increase plant height, emergence rate, growth promoters (i.e., indoleacetic acid, gibberellins, and cytokinins), and reduce endogenous inhibitors (abscisic acid (ABA) and phenol) in seed shells, enzymes involved in the synthesis of amino acids, lipids, and carbohydrates. Laser irradiation, particularly low – level laser therapy (LLLT), can stimulate cellular metabolism and enhance the activity of antioxidant enzymes such as superoxide dismutase (SOD). The primary mechanism involves the absorption of laser light by cytochrome c oxidase in the mitochondria, leading to increased ATP production and improved cellular energy status. As results, antioxidant defense systems become more active, allowing enzymes like SOD to neutralize harmful reactive oxygen species (ROS) and reduce oxidative stress. However, it is important to note that beneficial effects depend on Laser parameters such as Wavelength,intensity and exposure time [18, 20]. Based on recent evidence that laser bio-stimulation enhances germination, pigment biosynthesis, antioxidant defense, and metabolic activity in various plant species [21], we hypothesized that laser irradiation would significantly improve the growth, physiological traits, and secondary metabolite production of *Ocimum basilicum* L.

This study investigated the effects of diode laser irradiation (450 nm, blue; 650 nm, red; 100 mW; 5 and 10 min) on the growth, biochemical composition, phytohormones, essential oil content, enzyme activity, leaf anatomy, and molecular characteristics of *O. basilicum* L. seeds with the aim of the optimal wavelength and exposure duration to enhance plant performance and quality.

## Materials and methods

### Plant material, seed source, and taxonomic identification

The experiment was conducted in the greenhouse of the Agricultural Botany Department, Faculty of Agriculture, Cairo University, Giza, Egypt during the summer growing season of 2024/2025. Sweet basil (*Ocimum basilicum* L., Family: Lamiaceae), specifically Italian basil (Genovese type) with a characteristic mint-like aroma, was used in this study. Seeds were obtained from the Agricultural Museum, Ministry of Agriculture and Land Reclamation, Giza, Egypt, which provides authenticated cultivated plant material for research purposes. The seeds originated from cultivated plant material and were not collected from wild populations; therefore, no specific permissions were required for their use.

The taxonomic identity of *Ocimum basilicum* L., first described by Linnaeus (1753), is well established. The scientific name and taxonomic status were verified using internationally accepted taxonomic references. *Ocimum basilicum* L. is a herbaceous, widely cultivated species and is not listed as endangered or protected under national or international conservation regulations.

All experimental procedures involving plant material were conducted in accordance with institutional and national guidelines for experimental research on plants and complied with local legislation. The study is consistent with the principles of the Convention on the Trade in Endangered Species of Wild Fauna and Flora (CITES).

Sweet basil (*O. basilicum* L.) seeds were sown on March 13, 2024 in black plastic pots (30 cm diameter × 50 cm depth) containing a 2:1 (v/v) clay: sand mixture. Fertilization was performed according to standard recommendations for basil production, with NPK (20:20:20) applied at a rate of 2 g L⁻^1^ every 15 days starting three weeks after germination. Irrigation was carried out every two days to maintain soil at approximately 80% of field capacity throughout the experiment. Greenhouse temperature was maintained at 28 ± 3 °C during the day and 20 ± 2 °C at night, with relative humidity between 60–70% and a natural photoperiod averaging 13 ± 1 h light: 11 ± 1 h dark.

**Fig. 1:**
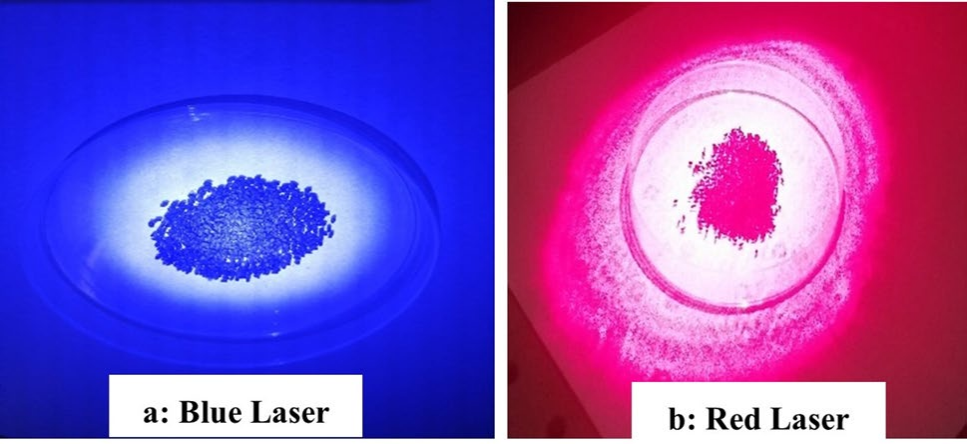
Irradiation of sweet basil seeds by (**a**) blue laser 450 nm, 100mW, (**b**) red laser 650 nm, 100mW

The Sweet Basil seeds (*O. basilicum* L.) were irradiated using a diode laser system. Treatments consisted of exposure to Red Laser (650 nm) and Blue Laser (450 nm) as shown in Fig. 1, each for durations of 5 and 10 min (denoted as RL5, RL10, BL5, and BL10, respectively). The laser source had an average output power of 100 mW.

To ensure uniform exposure across a batch of seeds, the laser beam was expanded to 5 cm spot size (beam spot area = ~ 19.63 cm^2^). The seeds were placed in a single layer at 25 cm from the laser aperture.

The total energy delivered per treatment was calculated as Power × Time, yielding 30 J for the 5-min treatments and 60 J for the 10-min treatments. The energy dose (fluence) was calculated as Total Energy/Beam Spot Area. Accordingly, the doses were,1.53 and 3.06 J/cm^2^ for the 5- and 10-min treatments, respectively.

In addition to the un-irradiated seeds which were considered as control plants, in order to study their effects on growth performance, physiological, biochemical, anatomical traits as well as essential oils determination and genetic molecular markers. The experiment was set up in a Randomized Complete Block Design with three replications. The experiment involved 15 pots with 5pots in each replicate. Irrigation was performed on a regular basis to maintain soil field capacity throughout the experiment.

### Plant analysis

#### Measurement of vegetative growth parameters

**Fig. 2:**
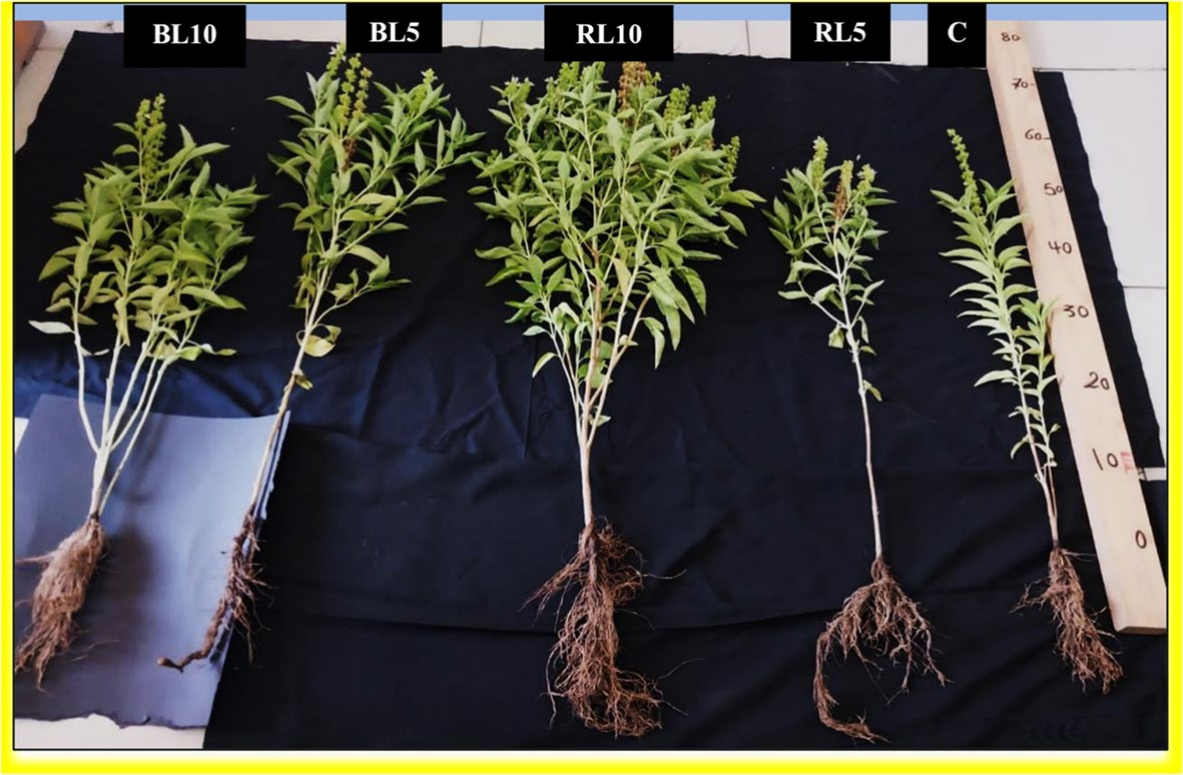
Comparative effects of laser irradiation at different exposure times on phenotypic changes of sweet basil plants. (C) untreated plants (control), (RL5) plant treated with red laser for 5 min, (RL10) plant treated with red laser for 10 min, (BL5) plant treated with blue laser for 5 min, (BL10) plant treated with blue laser for 10 min

A random sample of nine plants from each treatment (three plants from each replicate) was chosen for analysis. Morphological and anatomical characteristics were recorded after 110—days from sowing date in the first season. This age signifies the complete flowering stage. Plant height (cm), measured from the cotyledonary node to the plant^'^s highest point, main stem length (cm) was measured from the cotyledonary node up to the shoot apex, number of internodes of the main stem, number of branches per plant, number of leaves per plant, main root length (cm), and fresh and dry shoot weight (g) per plant were all measured (Fig. 2).

### Chemical analysis

#### Extract preparation

The fresh plant leaves were collected, dried, and powdered into fine powder. For sample preparation, 20 g of dried powder from each sample was extracted in triplicates with 70% ethanol at 25 °C for 24 h, then filtered and concentrated. The yield for each extract was calculated according to the following equation: The extraction yield (%) = (extract weight/plant sample weight) × 100. Each extract was tested for antioxidant activity using 2,2-Diphenyl-1-picrylhydrazyl (DPPH) and 2,2'-azino-bis (3-ethylbenzothiazoline-6-sulfonic acid) (ABTS) techniques, and the total phenolic content was determined.

#### Total phenolic content determination

The total phenolic contents (TPC) of five samples were determined by Folin-Ciocalteu assay, as reported by [22]. Gallic acid was employed as a standard (100–500 ppm). 100 µL of 1000 ppm extract was added to 750 µL of previously 10 folds diluted Folin-Ciocalteu solution and thoroughly mixed. After 5 min standing at room temperature, 750 µL of sodium carbonate (6%) was added. Mixtures were kept for 90 min then absorbance was measured at 725 nm using a UV/Vis spectrophotometer. The total phenolic content of each extract was estimated as in triplicates mg gallic acid equivalent per gram dry weight (GAE/g D.W.).

### Antioxidant activity

#### DPPH radical scavenging method

Blois [23] measured scavenging ability using the 1, 1-diphenyl, 2-picrylhydrazyl free radical (DPPH^•^). 100 µL sample of various concentrations (200, 400, 600, 800, and 1,000 ppm) was added to 900 µL of a 0.1 mmol DPPH methanolic solution. Methanol, 100 µL, was utilized as a control. After 30 min, the absorbance at 517 nm was measured. Ascorbic acid was determined as a standard antioxidant agent. The inhibition percentage was estimated as in the following equation:

$$\begin{aligned}&\mathrm{Inhibition}\;\mathrm{percentage}\;\left(\%\right)\\&\;=\;\left[\left(\mathrm{Ac}-\mathrm{As}\right)/\mathrm{Ac}\right]\times100\end{aligned}$$

In which Ac is the control absorbance and as is the sample absorbance.

The concentration (ppm) which inhibits 50% of DPPH^•^ was estimated as IC_50_.

#### ABTS radical scavenging assay

The radical scavenging activity of extracts against radical action (ABTS^+^) was determined using the procedures of Re [24]. The ABTS radical was created by reacting 7 mmol L^−1^ of ABTS solution with 2.45 mmol L^−1^ of potassium persulphate and leaving the mixture at room temperature for 16 h. In the time of usage, the ABTS solution was diluted with ethanol to an absorbance of 0.70 ± 0.02 at 734 nm. Each sample (0.2 ml) at varying concentrations (0–1.0 mg ml^−1^) was violently mixed with 2 ml of ABTS solution. After 6 min of processing, the absorbance at 734 nm was recorded. The ABTS scavenging effect was estimated as in triplicates the following formula:

$$\mathrm{Inhibition}\;\mathrm{percentage}\;\left(\%\right)\;=\;\left[\left(\mathrm{Ac}-\mathrm{As}\right)/\mathrm{Ac}\right]\times100$$

In which Ac is the control absorbance and as is the sample absorbance.

The concentration (ppm) which inhibits)50% (of ABTS radicals was estimated as IC_50_.

### Pigment analyses: assay for total carotenoids and chlorophylls a and b

Costache [25] determined the total carotenoids, chlorophylls a, and b. The samples were extracted in triplicates with acetone (500 mg of fresh leaves extracted with 10 ml of acetone), ground with a mortar and pestle, and homogenized. The samples were filtered and centrifuged at 5000 rpm for ten minutes. The absorbance was recorded at UV/VIS spectrophotometer at 470, 645 and 662 nm. The concentrations of pigments were calculated as follow:

$$\begin{aligned}&\mathrm{Chlorophyll}\;\mathrm a\;\left(\mathrm\mu/\mathrm g\;\mathrm{fresh}\;\mathrm{weight}\right)\\&\;\equiv\;\left[11.75\;\left({\mathrm A}_{662}\right)\;-\;2.350\;\left({\mathrm A}_{645}\right)\right]\times\mathrm V/\mathrm W\end{aligned}$$

$$\begin{aligned}&\mathrm{Chlorophyll}\;\mathrm b\;\left(\mathrm\mu/\mathrm g\;\mathrm{fresh}\;\mathrm{weight}\right)\\&\;=\;\left[18.61\;\left({\mathrm A}_{645}\right)\;-\;3.960\;\left({\mathrm A}_{662}\right)\right]\times\mathrm V/\mathrm W\end{aligned}$$

$$\begin{aligned}&\mathrm{Total}\;\mathrm{carotenoids}\;\left(\mathrm\mu/\mathrm g\;\mathrm{fresh}\;\mathrm{weight}\right)\;=\\&\;\left[1000\;\left({\mathrm A}_{470}\right)\;-2.270\;\mathrm{Chlorophyll}\;\mathrm a\;-\;81.4\;\mathrm{Chlorophyll}\;\mathrm b\;/227\right]\times\mathrm V/\mathrm W\end{aligned}$$

$$\begin{aligned}&\mathrm{Total}\;\mathrm{pigments}\;\left(\mathrm\mu/\mathrm g\;\mathrm{fresh}\;\mathrm{weight}\right)\\&\;=\;\mathrm{chlorophyll}\;\mathrm a\;+\mathrm{chlorophyll}\;\mathrm b\;+\mathrm{carotenoids}\end{aligned}$$

In which, A: absorbance at specific wavelength, V: final volume and W: weight of fresh tissue.

### Antioxidant enzymes assays

For 5 min, 500 mg of fresh leaves were homogenized in 1 ml K-phosphate buffer pH 6.8 containing 100 µL EDTA before being centrifuged at 10,000 rpm for 20 min at 40 °C. The supernatant was utilized to determine catalase (CAT) and glutathione peroxidase (GPx) enzyme activity. Catalase activity (unit/g) was measured in accordance with Aebi [26] using a colorimetric technique kit (catalogue number: CRL10517, Biodiagnositic Co., Egypt). The activity of GPx (mU/g) was measured in triplicates as described by Paglia and Valentino [27].

### Phytohormones analysis

#### *Ocimum basilicum* preparation for scanning by GC/MS/MS

Leaves were cleaned and air-dried for two days at room temperature in the shade, then ground into a coarse powder using a hand-grinding mill. A 10 g portion of the dried leaf powder was weighed and placed in a beaker containing 50 mL of absolute ethanol. The mixture was shaken for 1 h at room temperature using a mechanical shaker and then filtered through Whatman No. 1 filter paper. The residue was re-extracted twice under the same conditions, and the filtrates were combined. One microliter (1 μL) of the final extract was injected into the GC–MS/MS system after diluting 0.2 mL of the extract with two mL of ethanol.

#### *Ocimum basilicum* sample preparation for determine Gibberellic acid (GA3) and indole-3-acetic acid (IAA)

*O. basilicum* leaves were collected, freeze-dried, and homogenized ultrasonically in 70% acetone (40 ml/g dry weight) containing 100 mg Na ascorbate/L [28]. Extraction was carried out overnight at 4 °C. The acetone extract was filtered through Whatman No.1 paper covered with a single layer of Miracloth (Sigma-Aldrich) and evaporated under reduced pressure at 40 °C using a rotary evaporator. The residue was washed twice with distilled water, and the combined filtrates were acidified to pH 2.8 with 1 M H₃PO₄. The acidified fractions were passed through a 25 mm syringe filter and extracted three times with dichloromethane. The combined dichloromethane layers were evaporated to dryness and subjected to methylation [29].

### Determination of GA3 and IAA by GC–MS/MS

Gas chromatography (Agilent Technologies 7890 A) coupled with a mass-selective detector (MSD, Agilent 7000) was used to determine GA3 and IAA. Phenyl methyl polysiloxane (5%, HP-5 ms, Agilent), column (capillary, 30 m × 250 µm i. d. and 0.25 μm film thicknesses), and one ml per minute of helium was utilized as the carrier gas. The oven's program was (85 °C/2 min, rate of 40 °C/min per two min, 220 °C/one min at a rate of 30 °C/min, and 275 °C). Both the injector and detector had a temperature of 250 °C. One μL of volume was injected using a splitless mode technique. The mass has an interface temperature of (290 °C) and an ionization potential of 70 eV. Selected Ion Monitoring System (SIM) were used (97, 103, 129, 130, 157, and 175 m/z). Mass fragment at 77, 91, 115, 121, 136, 239, 281, and 328 m/z were used to determine the GA3.The oven's program alternated between 120 °C every two minutes, 30 °C every 2 min, and 280 °C every 0.4 min. Quantitative results were obtained by GC–MS/MS results and compared with GA3 and IAA standards.

### GC–MS/MS analysis program for scanning *Ocimum basilicum* compounds

The GC–MS/MS method was utilized to analyze the chemical composition of *O. basilicum*. Helium was employed as the carrier gas at a constant linear flow rate of one mL min⁻^1^. The oven temperature program was set as follows: an initial temperature of 55 °C (held for 3 min), followed by a rise to 280 °C at a rate of 11 °C min⁻^1^. The injector and detector temperatures were maintained at 220 °C. A one μL sample was injected in splitless mode. The ionization potential was set at 70 eV, and the interface temperature was maintained at 280 °C according to the mass spectrometer operating parameters. Mass spectra were recorded in scan mode (m/z 35–600). Compound identification was performed by comparing the obtained mass spectra with those in the NIST and WILEY libraries and by matching the fragmentation patterns with data reported in the literature [30].

### Leaf anatomy study

Sweet basil (*O. basilicum* L.) leaf samples were taken from the 4 node (the terminal end) leaves during vegetative growth. The specimens were gathered from the leaf between (mid-vein and the leaf margin) and fixed in formalin-acetic acid alcohol (FAA) utilized ethanol (70%). According to Johansen [31], the samples were dehydrated through a graded series of tert-butyl alcohol (TBA) before embedding in paraffin wax at (56 °C). The Transverse sections were cut at a thickness of (15–20 μm) using a rotary microtome (Model RM2245, Leica Microsystems). The paraffin wax was removed with xylol, after which the slides were stained with crystal violet- erythrosine before being mounted in Canada balsam [32]. A light microscope (Model BX51, Olympus Optical) was used to examine and photograph the selected sections. Each value represents three portions with three readings each.

### Molecular markers experiment

The Start Codon Targeted (SCoT) and Inter Simple Sequence Repeat -PCR (ISSR-PCR) were used as a molecular marker for detecting the genetic diversity among the *O. basilicum* plants after exposing radiation treatments. The SCoT and ISSSR analysis were carried out at the Agricultural Genetic Engineering Research Institute, Agricultural Research Center, Giza, Egypt. 110-day-old fresh leaves were collected from *O. basilicum* irradiated and un-irradiated plants (control), then kept at –80 °C for further DNA isolation steps.

### DNA extraction, SCoT and ISSR markers amplification

Total genomic DNA was extracted from leaves of *Ocimum basilicum* using a modified cetyltrimethylammonium bromide (CTAB) method as described by Doyle and Doyle [33], with modifications to improve DNA purity from aromatic and oil-rich tissues. Basil leaf tissue (100 mg) was ground in liquid nitrogen and homogenized then CTAB extraction buffer was added containing 2% (w/v) CTAB, 100 mM Tris–HCl (pH 8.0), 20 mM EDTA, 1.4 M NaCl, 1% (w/v) polyvinylpyrrolidone (PVP), and 0.2% β-mercaptoethanol. The mixture was incubated for 30 min at 65 °C, followed by extraction with chloroform: isoamyl alcohol (24:1 v/v). The aqueous phase was transferred, and DNA was precipitated with isopropanol and incubated at − 20 °C for 30 min. The resulting DNA pellet was washed with 70% ethanol, then dissolved in TE buffer (10 mM Tris–HCl, 1 mM EDTA, pH 8.0). To minimize contamination from phenolic compounds and essential oils, PVP and β-mercaptoethanol were included in the extraction buffer, as recommended for aromatic plant species [34]. DNA purity and concentration were assessed spectrophotometrically (A260/A280 ratio) and by electrophoresis on 1% agarose gel.

#### SCoT, ISSR markers amplification

The amplification reaction was carried out using 2 SCoT primers and 8 ISSR primers as shown in Table 1. Both SCoT and ISSR amplification reaction were composed as shown in Table 2 and the PCR reaction were processed following the thermal cycles of 94 ˚C/1 min as initial denaturation cycle, the annealing was performed at 50 °C for 1 min, followed by 72 ˚C for 45 s as extension, ended with 72 ˚C 10 min with a total number of 35 cycles. All PCR amplifications for both SCoT and ISSR assays were performed in three independent biological replicates per treatment. The PCR products, DNA Marker Ladder (1000 bp) were electrophoresis on 1% agarose gel, then visualized using the UV light and the band’s weigh and properties were analyzed by Gel Documentation System (BIO-RAD 2000).

**Table 1:** The Sequence of SCoT and ISSR primers and its details

Primer name	Primer sequence	Annealing Temp
SCoT-01	5'- ACGACATGGCGACCACGC −3'	49
SCoT-02	5'- ACCATGGCTACCACCGGC −3'	49
ISSR-03	5'- ACGACAGTGCGACCCACA −3'	50
ISSR-04	5'- ACCAGTGCTACCACCGCA −3'	50
ISSR-05	5'- CAATCGCTACCACTAGCG −3'	50
ISSR-06	5'- CAAGTGCTACCACTACAG −3'	50
ISSR-07	5'- ACAACGGCTACCACTGAC −3'	50
ISSR-08	5'- CAACAATCGCTACCACGT −3'	50
ISSR-09	5'- ACAATGGCTACCACTGCC −3'	50
ISSR-10	5'- ACATTGGCTACCACCAGC −3'	50

**Table 2:** The SCoT and ISSR Markers reaction contents

Content	SCoT analysis	ISSR analysis
Master mix with Taq polymerase (Sigma)	12.5 μl	12.5 μl
DNA template	1 μl (10 ng/μl)	1 μl (10 ng/μl)
Primers	2.5 μl (10 pcmol)	2.5 μl (10 pcmol)
distilled water	9 μl	9 μl

### Data scoring and statistical analysis

A randomized complete block design (RCBD) was applied, and each treatment was replicated in triplicate. The measured data on the morphological and anatomical properties of sweet basil plants were subjected to traditional methods of variance analysis, namely one way analysis of variance (ANOVA) as described by Snedecor and Cochran [35]. The least significant difference (LSD) test was calculated at the 0.05 probability level to compare treatment means. The results of chemical and phytohormones properties were assessed at 0.05 level of probability using the “COSTAT” statistical computer program which is based on a One-way analysis of variance ANOVA performed, followed by the student-Newman Keuls test. Differences were considered statistically significant at p < 0.05. The collected data on biochemical genetic markers were analyzed using the SPSS application System version 7 (SPSS Inc. 1997 SPSS Inc.3/97 standard version).

The size of the polymorphism was calculated in terms of percentage, using total number of polymorphic bands (binary data) obtained from SCoT and ISSR analysis. Correlation analysis to estimate the similarity between 4 controls vs. *Ocimum* treated samples The SCoT and ISSR bands were scored as 1 if present and 0 if absent. Analyze these markers to quantify the information they bring in distinguishing between *Ocimum* lines. The percentage of polymorphism (PPB) was calculated as described by Powell et al. [36]:

$$\begin{aligned}&\mathrm{PPB}\;\left(\%\right)\;=\\&\;\left(\mathrm{Number}\;\mathrm{of}\;\mathrm{polymorphic}\;\mathrm{bands}\;/\ \;\mathrm{Total}\;\mathrm{number}\;\mathrm{of}\;\mathrm{bands}\right)\\&\times100\end{aligned}$$

The tree diagram was constructed in similarity data clustered with the UPGMA (Unweighted Pair Group Method of Means) method. Source: GHPS, NDPHS Country files 7 (SPSS Inc. 1997 SPSS Inc3/97 standard version).

## Results and discussion

### Plant growth

Our results demonstrated the impact of 2 laser irradiation wavelengths under varying exposure times, on the growth performance of sweet basil plants (*O. basilicum* L.). The un-irradiated sweet basil plant was used as a control sample, some plant growth indicators covering, plant height, main stem length, number of leaves per plant, number of branches per plant, number of the main stem internode, main root length, shoot fresh weight and shoot dry weight were measured.

Our data presented in Table 3 demonstrates that exposure of sweet basil seeds to red and blue laser light at exposure time (5 and 10 min) stimulated and enhanced most of vegetative growth parameters as compared to those seeds without treatment (control). The most effective treatment was discovered from the exposure time of 10 min with the red laser, it achieved highest significant increments in plant height (cm), main stem length (cm), number of leaves per plant, number of branches per plant, number of the main stem internodes, main root length (cm), fresh and dry weight of shoot (g) by 28.07, 27.3, 68.3, 193.02, 34.5, 30.3, 126.5and 87.4% as compared to the control plants, respectively.

**Table 3:** Effect of Red and Blue laser irradiation at exposure time 5 and 10 min on the vegetative growth parameters of sweet basil plant (110- days old) in 1 st summer growing season of 2024/2025

Morphological Parameters	Treatments					L.S.D
	**Control (C)**	**RL5**	**RL10**	**BL5**	**BL10**	
Plant height (cm)	57.7 ± 3.34^b^	53.3 ± 5.43^b^	73.9 ± 0.58^a^	53.3 ± 9.16^b^	63.5 ± 2.24^b^	13.2
Main stem length (cm)	48.3 ± 3.52^b^	47.4 ± 5.13^b^	61.5 ± 0.74^a^	39 ± 4.87^c^	61.3 ± 1.01^a^	9.18
No. of leaves/plant	77.3 ± 2.45^c^	84.3 ± 8.95^b^	130.1 ± 23.7^a^	84.6 ± 1.16^b^	124.3 ± 19.5^a^	36.8
No. of branches/plant	4.3 ± 0.72^c^	5.6 ± 1.92^c^	12.6 ± 3.00^a^	7.1 ± 1.09^b^	9.0 ± 1.04^b^	4.5
No. of the main stem internodes	17.1 ± 0.92^b^	19.1 ± 1.20^b^	23.0 ± 1.25^a^	17.6 ± 0.66^b^	20.1 ± 1.20^b^	2.75
Main root length (cm)	14.25 ± 1.70^b^	17.08 ± 1.02^a^	18.58 ± 0.41^a^	15.35 ± 0.58^b^	17.33 ± 0.58^a^	2.51
Shoot fresh weight (g)	23.14 ± 3.54^d^	35.5 ± 11.21^c^	52.42 ± 9.89^a^	38.79 ± 9.11^c^	44.31 ± 1.90^b^	20.59
Shoot dry weight (g)	6.30 ± 1.77^b^	4.33 ± 0.35^c^	11.81 ± 5.45^a^	8.21 ± 2.83^b^	6.79 ± 2.37^b^	7.83

^*^C: untreated plants (control), RL 5: plant treated with red laser for 5 min, RL 10: plant treated with red laser for 10 min, BL 5: plant treated with blue laser for 5 min, BL 10: plant treated with blue laser for 10 min

^*^The same superscript letters within a row designate there was no significant difference according to the Duncan multiple comparison test at (*p ≤* 0.05) correspondingly

^*^Each value is the mean of three replicates over one season (summer growing season). Means ± values indicated to standard error (± SE)

A similar pattern was observed from the plants treated by the blue laser at exposure time (10 min) over those of un- irradiated plants(control), it recorded the maximum values in all previous mentioned vegetative growth characters by 10.05%, 26.9%, 60.8%, 109.3%, 17.5%, 21.6%, 91.4% and 7.7% for plant height (cm), main stem length (cm), number of leaves per plant, number of branches per plant, number of the main stem internodes, main root length (cm), fresh and dry weight of shoot (g) over than control, respectively as shown in Table 3.

These findings are in accordance with Prysedsky and Kozlova [7], who examined the impact of monochromatic coherent light, produced by 100 mW LED lasers with wavelengths of 635 and 405 nm on growth parameters of a few Poaceae family plant species, including *Zea mays* L., *Avena sativa* L., and *Triticum aestivum* L. and found that the examined plants' germination readiness and ability were only likely to enhance when red and blue light were combined for ten seconds. Plant growth metrics improved significantly (10.4–124, 5%) based on the irradiation's dose and spectral composition. Complex (red & blue) light laser irradiation of seeds radiation and red light for ten seconds are the most effective and have an impact on the concentration of chlorophyll a and chlorophyll b.

Similarity, AlSalhi [19] reported that He–Ne laser (633 nm) and an argon laser (514.5 nm) at low power significantly improved the growth and germination of wheat seeds *(Triticum aestivum L*.).

According to Metwally [37], exposure of *Celosia argentea* to helium–neon laser rays for two or three minutes resulted in significant increases in plant height, leaf number per plant, stem diameter, root length, and days from planting to flowering, compare to the control.

Plant growth is known to be controlled by several enzymes and hormones, particularly gibberellic acid (GA₃) and cytokinin. The GA3 primally induces cell elongation, in addition to inducing proteolytic enzyme formation and increase of auxin level. Consequently, helium–neon laser–treated plants showed stimulated cell elongation and plant height compare to untreated controls, leading to longer internodes, increased branching, and a greater number of leaves per plant [38].

Recent studies have provided quantitative evidence that laser irradiation can significantly enhance various physiological and biochemical traits in plants. For instance, exposure to red or blue laser light has been shown to increase seed germination rate, chlorophyll concentration, and shoot biomass by 15–40%, depending on the wavelength and exposure duration [39].

Similarly, Kazemzadeh-Bene et al. [40] reported that laser treatment induced higher antioxidant enzyme activities and improved photosynthetic performance in red-fleshed apple cultures.

Furthermore, Almuhayawi et al. [41] confirmed that laser irradiation stimulated pigment accumulation and enhanced oxidative stress tolerance in *O. basilicum* and related *Lamiaceae* species. These findings highlight the quantitative physiological responses of plants to laser bio-stimulation and support its potential as an eco-friendly growth-enhancement technology.

### Effect of laser irradiation on total phenolic content and antioxidant activity (DPPH and ABTS assays)

Total phenolic content and the antioxidant activity of the *O. basilicum* exposed to laser radiation were assessed for studying the effects of different laser irradiation wavelengths with different exposure times. The results showed in Table 4 indicated that the exposure to blue laser radiation for 10 min (BL10 sample) significantly increased the total phenolic content to 34.01 ± 0.21 mg GAE/g dried sample compared with non-exposed sample (32.33 ± 0.14 mg GAE/g dried sample).

**Table 4:** The effect of seeds laser irradiated at two wavelengths with different exposure times on extraction yield (g/100 g), total phenolic content (mg GAE/g dried sample) by Folin –Ciocalteu method and antioxidant activity by DPPH and ABTS methods of *Ocimum basilicum* samples

Treatments	Extract yield (g/100 g)	Total phenolic content (mg GAE/g dried sample)	Antioxidant activity * IC_50_ (μg/ml)	
			DPPH method	ABTS method
Control	20.53 ± 0.09^c^	32.33 ± 0.14^b^	710.60 ± 3.80^a^	844.15 ± 3.42^a^
RL5	11.29 ± 0.15^e^	18.75 ± 0.06^e^	668.77 ± 2.60^b^	624.75 ± 1.38^c^
RL10	12.32 ± 0.15^d^	24.30 ± 0.10^d^	648.25 ± 2.45^c^	716.68 ± 1.81^b^
BL5	28.82 ± 0.17^a^	28.99 ± 0.08^c^	639.13 ± 1.96^d^	526.70 ± 6.13^d^
BL10	27.06 ± 0.12^b^	34.01 ± 0.21^a^	596.90 ± 1.45^e^	433.40 ± 0.33^e^
Vit. C	-	-	29.13 ± 0.15^f^	25.64 ± 0.11^f^

^*^Control: untreated plants, RL 5: plant treated with red laser for 5 min, RL 10: plant treated with red laser for 10 min, BL 5: plant treated with blue laser for 5 min, BL 10: plant treated with blue laser for 10 min

^*^Values are mean ± SEM. *IC50 = concentration required to inhibit 50% of DPPH radical (DPPH method), ABTS radical (ABTS method)

The antioxidant activity of *O. basilicum* samples determined by different methods showed that radiated samples (RL5, RL10, BL5, BL10) emerged significantly improving of scavenging abilities, decreasing of IC_50_ values, against DPPH (668.77 ± 2.60^b^, 648.25 ± 2.45^c^, 639.13 ± 1.96^d^ and 596.90 ± 1.45^e^ μg/ml, respectively) and ABTS radicals (624.75 ± 1.38^c^, 716.68 ± 1.81^b^, 526.70 ± 6.13^d^ and 433.40 ± 0.33^e^ μg/ml, respectively).

This result indicated that the increasing of phenolic compounds content contributed to oxidants removal. The result agreed with Dudonne [42], who reported that the high phenolic content contributes strongly to antioxidant properties of different plant samples. That is may be due to the chemical structure of plant phenols rises its antioxidant characteristics.

To assess the antioxidant efficiency of different samples obtained after seeds irradiation, the collected samples applied in different concentrations for evaluation of its scavenging activity by DPPH and ABTS methods in comparison with vitamin C as a natural antioxidant material.

The results shown in Tables 4 and 5 indicated that the radical scavenging activities of radiated plants ranged in average from 45.98 ± 0.28 to 52.31 ± 0.14 and 44.12 ± 0.13 to 71.15 ± 0.08% by DPPH and ABTS methods, respectively. In DPPH assay, results revealed that the antioxidant activity of blue radiated samples gave the highest effect at 10- and 5-min exposure times (80.40 ± 0.27 and 77.93 ± 0.13%, respectively) at 1000 ppm and (68.55 ± 0.08 and 59.12 ± 0.14%, respectively) at 800 ppm while vitamin C showed 95.44 ± 0.11and 95.23 ± 0.04% at 1000 ppm and 800 ppm, respectively.

**Table 5:** Antioxidant activity (%) of Ocimum basilicum samples extracts against DPPH radicals at different concentrations

**Conc. (µg/ml)**	Antioxidant** activity (%)**						**LSD (0.05)**
	Control	**RL5**	RL10	BL5	**BL10**	Vitamin** C**	
200	7.77 ± 0.53^d^	11.86 ± 0.39^c^	7.93 ± 0.18^d^	28.85 ± 0.08^b^	29.41 ± 0.08^b^	88.55 ± 0.25^a^	0.93
400	41.89 ± 0.10^b^	40.44 ± 0.30^c^	41.57 ± 0.20^b^	37.38 ± 0.13^d^	34.01 ± 0.13^e^	92.91 ± 0.19^a^	0.57
600	57.17 ± 0.26^b^	50.47 ± 0.31^d^	55.04 ± 0.71^c^	43.57 ± 0.33^f^	49.19 ± 0.17^e^	94.40 ± 0.19^a^	1.15
800	62.23 ± 0.23^d^	57.59 ± 0.71^f^	64.71 ± 0.10^c^	59.12 ± 0.14^e^	68.55 ± 0.08^b^	95.23 ± 0.04^a^	0.96
1000	68.68 ± 0.10^e^	69.55 ± 0.35^d^	66.71 ± 0.06^f^	77.93 ± 0.13^c^	80.40 ± 0.27^b^	95.44 ± 0.11^a^	0.61
Mean	47.55 ± 0.23^d^	45.98 ± 0.28^e^	47.19 ± 0.24^d^	49.37 ± 0.16^c^	52.31 ± 0.14^b^	93.31 ± 0.15^a^	0.64

^*^Control: untreated plants, RL 5: plant treated with red laser for 5 min, RL 10: plant treated with red laser for 10 min, BL 5: plant treated with blue laser for 5 min, BL 10: plant treated with blue laser for 10 min

^*^Values are mean ± SEM. The mean values with different superscript letter within a row indicate significant difference (at *p ≤* 0.05)

This result indicated that the blue radiation exposed samples have more potent effect on DPPH radical than the red radiation exposed samples. The results shown in Tables 5 and 6 indicated that the radical scavenging activities of radiated plants ranged in average from 45.98 ± 0.28 to 52.31 ± 0.14 and 44.12 ± 0.13 to 71.15 ± 0.08% by DPPH and ABTS methods, respectively.

**Table 6:** Antioxidant activity (%) of Ocimum basilicum samples extracts against ABTS radicals at different concentrations

Conc. (µg/ml)	Antioxidant activity (%)						LSD (0.05)
	**Control**	RL5	RL10	**BL5**	BL10	Vitamin** C**	
200	23.21 ± 0.12^b^	7.83 ± 0.07^e^	3.82 ± 0.05^f^	15.57 ± 0.11^d^	18.96 ± 0.31^c^	94.82 ± 0.20^a^	0.52
400	28.89 ± 0.23^d^	17.06 ± 0.14^f^	22.64 ± 0.11^e^	43.74 ± 0.34^c^	52.86 ± 0.04^b^	97.06 ± 0.25^a^	0.64
600	36.49 ± 0.17^d^	33.56 ± 0.21^e^	35.81 ± 0.26^d^	58.63 ± 0.39^c^	90.45 ± 0.22^b^	97.60 ± 0.14^a^	0.76
800	41.54 ± 0.29^f^	56.74 ± 0.11^e^	67.88 ± 0.18^d^	81.93 ± 0.13^c^	94.19 ± 0.04^b^	97.93 ± 0.05^a^	0.49
1000	58.94 ± 0.10^f^	79.57 ± 0.08^e^	90.44 ± 0.07^c^	85.15 ± 0.26^d^	99.28 ± 0.30^a^	98.15 ± 0.05^b^	0.53
Mean	37.81 ± 0.17^f^	38.95 ± 0.12^e^	44.12 ± 0.13^d^	57.00 ± 0.24^c^	71.15 ± 0.08^b^	97.11 ± 0.13^a^	0.47

^*^Control: untreated plants, RL 5: plant treated with red laser for 5 min, RL 10: plant treated with red laser for 10 min, BL 5: plant treated with blue laser for 5 min, BL 10: plant treated with blue laser for 10 min

^*^Values are mean ± SEM. The mean values with different superscript letter within a row indicate significant difference (at *p ≤* 0.05)

In ABTS assay, results revealed that the antioxidant activity of blue radiated samples gave the highest effect at 10 min exposure time (BL10) in comparison with vitamin C followed by RL10, BL5, RL5 and control (99.28 ± 0.30, 98.15 ± 0.05, 90.44 ± 0.07, 85.15 ± 0.26, 79.57 ± 0.08 and 58.94 ± 0.10%, respectively) at 1000 ppm while at 800 ppm vitamin C followed by BL10, BL5, RL10, RL5 and control (97.93 ± 0.05, 94.19 ± 0.04, 81.93 ± 0.13, 67.88 ± 0.18, 56.74 ± 0.11 and 41.54 ± 0.29%, respectively) at 800 ppm.

These findings revealed that the laser wavelength was more effective than the exposure time at 800 ppm (lower concentration) while at 1000 ppm (higher concentration), the exposure time was more effective than laser wavelength. These concentration differences' effects may be due to the laser wavelengths which caused induction of plant cells to produce different antioxidant compounds with different scavenging rates.

The obtained results reported that blue irradiated plant extract after 10 min exposure (BL10) recorded significantly highest antioxidant ability in both DPPH and ABTS radical assays followed by BL5, RL10 and RL5 with mean of inhibition percentage of 52.31 ± 0.14, 49.37 ± 0.16, 47.19 ± 0.24, 45.98 ± 0.28 respectively in DPPH method and 71.15 ± 0.08, 57.00 ± 0.24, 44.12 ± 0.13 and 38.95 ± 0.12 respectively in ABTS method**.**

These results are in contrast with Taie, [43] who stated that the red laser irradiation has positively affected *Sequoia sempervirens* plantlet’s effect on DPPH radical scavenging activity higher than the blue laser irradiation of 62.50 and 57.2% respectively. This difference may be due to the different responses of two plants against irradiation wavelengths or the different ages of plants in which samples were collected.

These results are in contrast with our results which recorded the highly potential effect of blue laser irradiation on *O. basilicum* than red laser. This difference may be due to the different responses of two plants against irradiation wavelength or the different ages of plants in which samples were collected.

The recorded results indicated that the antioxidant activity of different irradiation wavelengths possessed various effects depending on the wavelength and exposure period, in addition to the phenolic quantity and composition which contribute in response to laser irradiations.

### Pigment analyses: total carotenoids and chlorophylls a and b

Evaluation of photosynthetic pigments was conducted for studding the effect of two wavelengths of laser radiation on the photosynthesis process which indirectly affecting germination and growth processes. The results shown in Table 7 revealed increasing in chlorophyll and total carotenoids contents as a result of radiation exposure in comparison with unexposed seeds.

**Table 7:** Effect of two wavelengths of laser irradiation at different exposure times on Ocimum basilicum photosynthetic pigments content, chlorophyll a, b and total carotenoids content (µg/g) fresh weight, and activities of glutathione peroxidase (GPx) and catalase (CAT) Enzymes

Treatments	**Chlorophyll a (µg/g) (A)**	**Chlorophyll b (µg/g) (B)**	**Total carotenoids (µg/g fresh weight) (C)**	**Total pigments (A + B + C)**	**GPx (**mU**/g)**	**CAT (unit/g)**
Control	18.20 ± 0.63^e^	15.60 ± 0.05^d^	20.26 ± 0.15^b^	54.07 ± 0.77^e^	0.20 ± 0.01^d^	1.43 ± 0.02^d^
RL5	47.36 ± 0.18^d^	25.70 ± 0.37^a^	17.19 ± 0.01^c^	62.86 ± 0.48^d^	0.22 ± 0.01^d^	1.45 ± 0.03^d^
RL10	28.63 ± 0.17^a^	25.55 ± 0.35^a^	15.46 ± 0.06^d^	88.37 ± 0.50^b^	0.64 ± 0.01^b^	1.71 ± 0.02^b^
BL5	32.80 ± 0.34^c^	22.98 ± 0.17^c^	11.76 ± 0.10^e^	67.53 ± 0.57^c^	0.38 ± 0.14^c^	1.55 ± 0.02^c^
BL10	43.91 ± 0.60^b^	23.81 ± 0.12^b^	22.64 ± 0.22^a^	90.35 ± 0.72^a^	0.70 ± 0.02^a^	1.80 ± 0.01^a^
**LSD (0.05)**	1.35	0.78	0.41	1.93	0.04	0.06

^*^Control: untreated plants, RL 5: plant treated with red laser for 5 min, RL 10: plant treated with red laser for 10 min, BL 5: plant treated with blue laser for 5 min, BL 10: plant treated with blue laser for 10 min

^*^Values are mean ± SEM. The mean values with different superscript letter within a column indicate significant difference (at *p ≤* 0.05)

Ten minutes blue radiation exposed samples showed increasing of carotenoids content compared with unexposed sample (22.64 ± 0.22 and 20.26 ± 0.15 µg/g fresh weight, respectively), on the other hand, ten minutes red radiation exposed sample possessed the highest contents of Chlorophyll a and b (28.63 ± 0.17 and 25.55 ± 0.35a µg/g, respectively). These results indicated that the determined total pigments contents of* O. basilicum* are laser radiation wavelength and exposure time dependent.

This result is in concise with Almuhayaw [21] who mentioned that photosynthetic pigments levels were enhanced with the effect of laser light and Taie [43] who determined the laser significant increase effect on the concentration of chlorophyll a, chlorophyll b and total chlorophyll, as well as on their photosynthetic activities.

The results are in agreement with [44, 45] who indicated that the specific wavelengths of laser light that is absorbed by phytochromes stimulate the conversion of light energy to chemical energy which finally lead to production of bioactive compounds such as phenolics photosynthetic pigments and antioxidant phytochemicals.

### Antioxidant enzymes activities

Antioxidant enzymes function synergistically to protect plant cells from oxidative damage induced by environmental stresses. The activities of glutathione peroxidase (GPx) and catalase (CAT) Enzymes were determined for assessment of the *O. basilicum* ability to scavenge ROS produced from laser radiation exposure. The results shown in Table 7 revealed that blue laser radiation stimulate both GPx and CAT activities more than red laser compared to control at the same exposure time.

Moreover, the increasing of GPx and CAT enzymes activities were proportionally related to the exposure time of both blue and red laser wavelengths. The blue laser exposure for 10 min sample showed the highest effect on GPx and CAT activities with 0.70 ± 0 0.02 mU/g and 1.80 ± 0.01 unit/g, respectively compared with unexposed sample with 0.20 ± 0.01 mU/g and 1.43 ± 0.02 unit/g, respectively.

This result is in consistent with Kazemzadeh-Bene [40] mentioned that laser irradiations effects on red-fleshed apple cell suspension were showed laser type-dependent effects which induced a significant increasing in CAT activity with decreasing in ROS production. The results revealed that both enzymatic (GPx and CAT) and non-enzymatic compounds (phenolic compounds carotenoids) of *O. basilicum* are used in plant defenses against laser irradiation oxidative effects.

These results proved that the laser radiation stimulate plant cells by using coherent, monochromatic light to trigger a complex chain of biochemical and physiological responses. These responses involve absorbing and storing light energy, increasing of stress resistance against produced reactive oxygen species (ROS) which have emerged as a multifunctional signaling molecules that modulate diverse stresses.

That led to increasing of total phenolic content antioxidant activity carotenoids and antioxidant enzymes activities such as catalase and glutathione peroxidase as indicators for increasing of laser stress resistance. That occurs by interacting with plant photoreceptors, influencing gene expression, regulating hormones, and initiating changes in metabolic pathways, such as photosynthesis which indicated by increasing of chlorophylls a and b [46].

### Determination of phytohormones (gibberellic acid and indole-3-acetic acid)

**Fig. 3:**
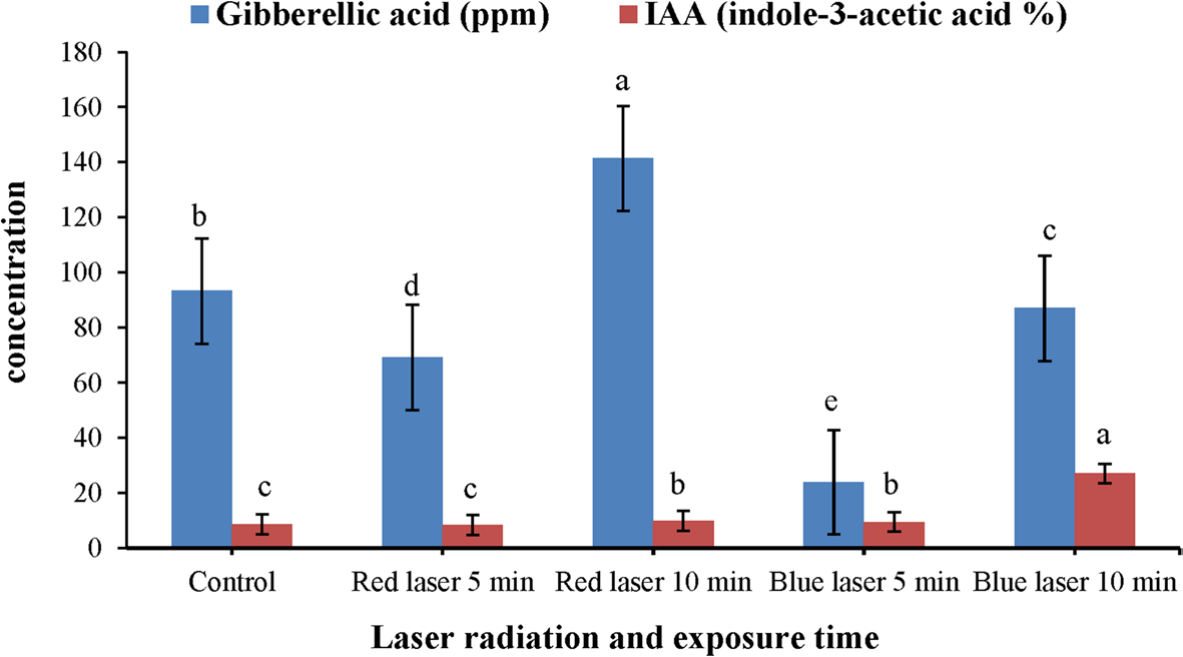
Effect of red laser (at wavelength 650 nm) and blue laser (at wavelength 450 nm) for 5- and 10-min power 1000 ml of sweet basil (*Ocimum basilicum* L.) on Gibberellic acid and indole-3-acetic acid. *Data are represented by means ± standard errors. Different small letters (a, b, c, d, e) above bars indicate significant differences between means at *p ≤* 0.05

Laser irradiation significantly influenced phytohormone levels in *O. basilicum* plants. The concentrations of gibberellic acid)GA3 (and indole-3-acetic acid (IAA) are summarized in Fig. 3. The findings demonstrated a significant increase in GA3 and IAA relative tocontrol group (*P* < 0.05). When compared to all other samples, the highest GA3 levels was observed in the red laser at 10 min (141.35 ppm), while the highest IAA (27.07%) was observed under blue laser for the same duration.

Our findings agree with Cope [47] who revealed that red light stimulates the enzymes for GA₃ biosynthesis through activation of diterpenoid pathway enzymes. While blue light stimulates enzymes that auxin biosynthesis and cytokinin regulation via photoreceptor-mediated signaling [48]. The observed hormonal modulation may therefore result from wavelength-specific activation of phytochromes and cryptochromes, which trigger downstream transcriptional regulation of hormone-related genes.

Matsuo [49] showed that level of bioactive GA was significantly higher in seedlings under red LED treatment as opposed to other light conditions.

Furthermore, recent studies suggest that laser-induced hormonal changes are linked with reactive oxygen species (ROS)-mediated signaling, which modulates hormonal balance, stress responses, and gene expression [50, 51]. This interaction between photoreceptor activation, ROS signaling, and hormonal biosynthesis likely contributes to the enhanced growth, elongation, and overall plant vigor observed under laser treatment.

### Chemical composition of sweet basil essential oil by (GC–MS/MS analysis)

The study identified a total of 53 different components contain 23 components of phenols and flavonoids and 30 components of essential oils which are provided in Table 8. Results revealed that red laser increased essential oils like (Eucalyptol, Eugenol, Caryophyllene) and flavonoid like (3,4',5-Trihydroxy-3',7-dimethoxyflavone, Isoflavone, 3',5,7-trihydroxy-4'-methoxy, m-Cresol, Quercetin 3,5,7,3',4'-pentamethyl ether). While essential oils like (Sabinene hydrate, Longiborneol, γ-Muurolene) and flavonoid like (3',4',5',5,6,7-Hexamethoxyflavone, Quercetagetin, 7,8,4'-Trimethoxyisoflavone, Kaempferol) were decreased.

**Table 8:** Effect of Read and blue laser in chemical compounds in sweet basil (Ocimum basilicum L.) plant

No	R.T	**Name**	C	RL 5	RL 10	BL 5	BL 10
1	4.051	7,4’-Homoisoflavane	1.54	0.69	1.54	1.43	1.27
2	5.199	Guaiol	0.78	0.59	0.34	0.58	0.43
3	6.294	cis-Sesquisabinene hydrate	0.49	0.23	0.35	0.47	0.39
4	6.364	Isoflavone, 6,7-dimethoxy-	0.44	0.17	0.34	0.31	0.34
5	6.959	Eucalyptol	1.94	3.59	1.98	1.93	1.15
6	7.094	Sabinene hydrate	0.43	0.21	0.41	0.41	0.2
7	7.402	β-Terpineol	0.42	0.34	0.63	0.37	0.45
8	7.754	Linalool	2.71	4.61	4.58	3.07	1.48
9	8.394	Camphor	0.51	0.25	0.34	0.79	0.57
10	9.071	Estragole	1.2	1.47	0.51	1.27	1.34
11	10.03	Isobornyl acetate	2.13	1.55	0.67	1.97	0.19
12	10.375	7-Mercapto-4-methylcoumarin	0.63	2.72	0.82	1.06	0.39
13	10.666	α-Santalol	0.4	0.6	0.37	1.01	0.51
14	11.023	Eugenol	5.01	1.35	5.87	2.03	2.64
15	11.264	Caryophyllene	1.08	6.12	0.8	0.77	1.11
16	11.732	α-Cedrene	2.65	2.32	3.19	1.66	1.73
17	11.998	Humulene	0.68	0.37	0.64	0.94	0.34
18	12.269	γ-Muurolene	1.21	0.66	0.98	0.97	0.47
19	12.601	Copaene	0.83	1.33	0.98	1.08	0.87
20	13.307	Ledene	0.57	1.1	1.09	0.23	0.19
21	13.331	Sinapic acid	1.34	0.43	0.56	0.25	1.02
22	13.897	Clovene	1.73	1.73	2.97	2.64	1.53
23	15.119	3,4’,5-Trihydroxy-3’,7-dimethoxyflavone	0.66	0.85	0.87	0.61	0.31
24	15.501	Phytol	0.8	0.23	0.51	0.95	0.58
25	15.738	3’,4’,7-Trimethylquercetin	0.91	0.6	0.64	0.44	0.58
26	15.878	Levomenthol	1.06	1.05	0.79	0.82	0.91
27	16.247	Kaempferol	1.18	0.92	0.51	0.91	0.7
28	16.649	Hexa-hydro-farnesol	0.44	0.97	1.89	0.95	0.89
29	16.801	Isoflavone, 3’,5,7-trihydroxy-4’-methoxy-	2.89	5.87	5.1	6.2	5.11
30	17.002	Flavone, 5-hydroxy-3,3’,4’,6,7-pentamethoxy-	0.68	1.78	2.04	0.83	2.96
31	17.047	m-Cresol	0.34	0.81	1.81	0.79	4.16
32	17.592	7,8,4’-Trimethoxyisoflavone	0.54	0.45	0.25	0.5	0.78
33	17.781	Citronellol	5.14	4.75	3.2	9.21	4.8
34	18.068	4’,5,7-Trihydroxy 3,6,8-trimethoxyflavone	0.49	1.91	0.38	0.68	0.43
35	18.199	Isolongifolol	1.02	4.81	3.64	2.94	3
36	18.269	Ledane	8.17	11.51	9.71	15.74	16.36
37	18.457	Kaempferol 3,7,4’-trimethyl ether	0.97	2.92	1.11	2.02	1.78
38	18.708	Quercetagetin	0.89	0.17	0.57	1.17	0.82
39	19.552	Longiborneol	0.42	0.39	0.22	0.54	0.66
40	19.561	Methyl cinnamate	0.49	1.46	0.36	1.08	0.65
41	19.991	8-Hydroxy-6,7-dimethoxycoumarin	0.42	2.43	0.29	0.27	0.3
42	20.143	7,8-Dimethoxy-4-methylcoumarin	0.51	1.56	0.26	0.34	0.36
43	20.282	5-Hydroxy-3’,4’,5’,6,7,8-hexamethoxyflavone	0.49	2.57	0.36	0.8	0.75
44	20.779	5,7,3’,4’,5’-Pentamethoxyflavone	0.46	1.55	0.47	1.27	1.06
45	20.815	Quercetin 3,5,7,3’,4’-pentamethyl ether	0.79	1.98	2.05	5.12	5.55
46	21.267	.6,7,8-Trimethoxycoumarin	0.4	6.65	3.56	0.85	1.24
47	22.271	Salicylic acid β-D-O-glucuronide	0.98	1.33	0.99	0.6	1.29
48	22.714	3,7,8,2’-Tetramethoxyflavone	0.47	2.01	0.87	1.09	0.88
49	22.948	5,7,2’-Trimethoxyflavone	1.22	1.95	1.93	1.4	2.44
50	23.108	Squalane	0.46	1.66	2.06	3.03	4.38
51	23.256	Luteolin 6,8-c-diglucoside	14.77	0.84	1.54	0.29	0.89
52	23.346	3’,4’,5’,5,6,7-Hexamethoxyflavone	22.46	0.25	1.05	1.02	1.25
53	23.658	7-epi-cis-sesquisabinene hydrate	0.74	1.39	21.03	12.3	15.52

^*^C: untreated plants (control), RL 5: plant treated with red laser for 5 min, RL 10: plant treated with red laser for 10 min, BL 5: plant treated with blue laser for 5 min, BL 10: plant treated with blue laser for 10 min

^*^The identified compounds accounted for 100% of the total essential oil composition

We noted that blue Laser decreased some terrapins like Eucalyptol, Guaiol, cis-Sesquisabinene hydrate, Sabinene hydrate, Isobornyl acetate, Eugenol, α-Cedrene, while increased Isoflavone, 3', 5, 7-trihydroxy-4'-methoxy-, Ledane, Squalane and 7-epi-cis-sesquisabinene hydrate.

Our results agree with Semenova [52] who found that UV-treatments (280–400) nm decreased sabinene and increased linalool. Ismail [53] analyzed that *O. basilicum* by GC–MS/MS and discovered that it contains Linalool, Eugenol, Caryophyllene and Camphor and that agree with our results. Gebrehiwot [54] revealed that *O. basilicum* leaves contain essential oils like (Estragole, Eugenol, Isocaryophillene), Flavonoids and Phenols and that agree with our results.

We noted that Caryophyllene in red laser 10 min decreased (0.8%) compared with control (1.08%) that due to higher concentration in GA3 and that agree with Hazzoumi [55] who found GA3 does not have a great effect in essential oil yield. We concluded that the highest concentration of GA3 and IAA result form effect blue and red laser at 10 min that have positive effect in increasing Linalool and Eugenol levels compare to control that agree with Nazmy [56].

The results revealed increasing of coumarins at 5 min exposure of red laser (7-Mercapto-4-methylcoumarinꓹ 8-Hydroxy-6,7-dimethoxycoumarinꓹ 7,8-Dimethoxy-4-methylcoumarin and 6,7,8-Trimethoxycoumarin). These results proved that the laser irradiation abiotic stress can influence the expression of biosynthetic genes and accumulation of coumarin in plants to cope with the oxidative stress induced by stress factors exposure to adapt with the environmental changes [57].

### Leaf anatomy study

**Fig. 4:**
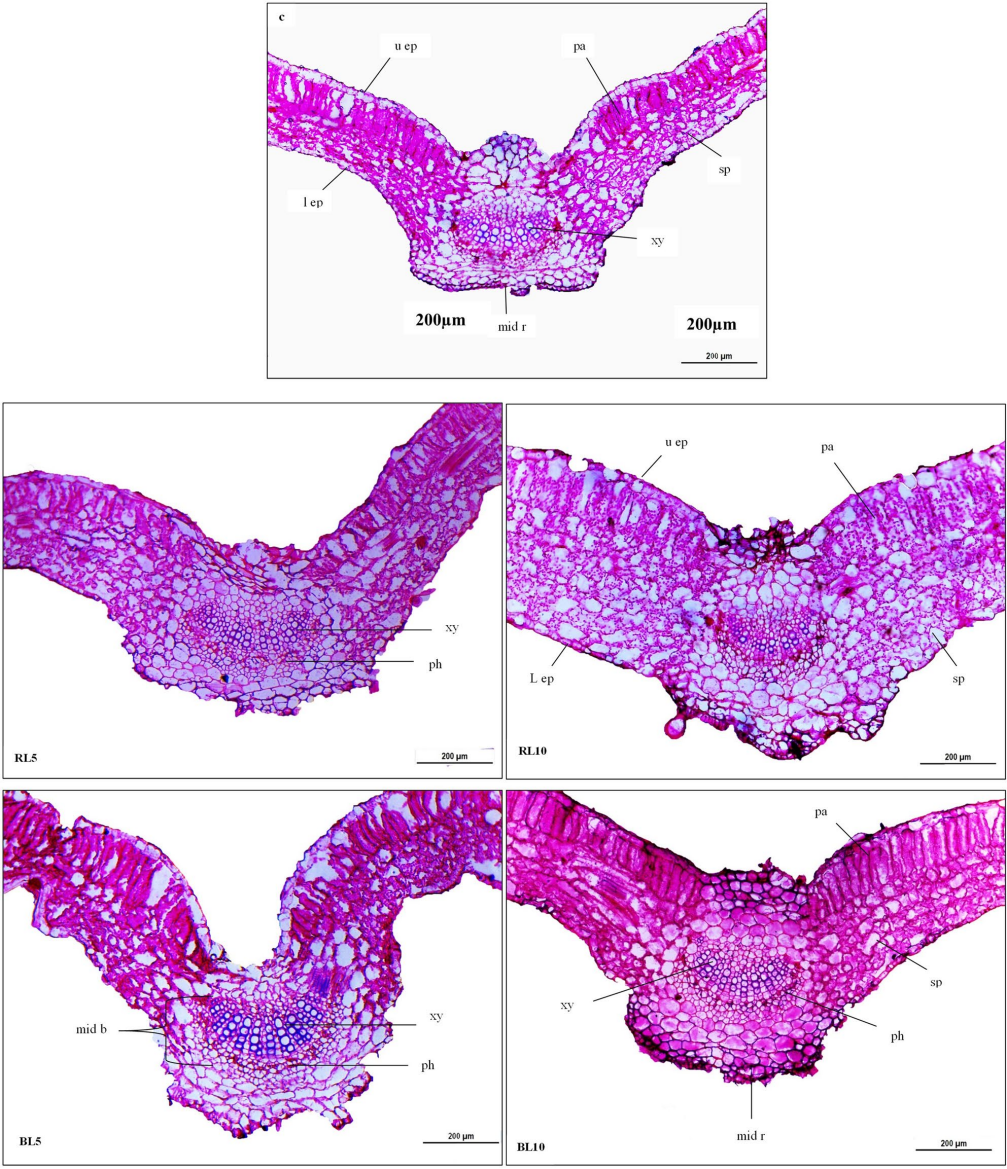
Transverse sections through the leaf blade of the fourth node developed on the median portion of the main stem of sweet basil plants as treatments by RL5, RL10, BL5, BL10 and Control in 2024 (first) summer season (200x). Where: up ep = upper epidermis, l ep = lower epidermis, pa = palisade parenchyma, sp = spongy parenchyma, mid b = midvein bundle, mid r = midrib region, xy = xylem tissue, ph = phloem tissue. (C) untreated plants (control), (RL5) plant treated with red laser for 5 min, (RL10) plant treated with red laser for 10 min, (BL5) plant treated with blue laser for 5 min, (BL10) plant treated with blue laser for 10 min

The objective of this study was to investigate the internal structure characteristics of sweet basil leaves that responded most significantly to red and blue laser irradiation at wavelengths (650 nm and 450 nm) for 5 and 10 min, respectively, as compared to control plants. Table 9 show microscopic counts and measurements of specific histological characteristics in cross-sections of sweet basil leaves. Similarly, microphotographs illustrating such treatments are shown in Fig. 4.

**Table 9:** Effects of Red and blue lasers on the histological characteristics (µm) of the sweet basil leaves developed on the median portion of the main stem of sweet basil plant

**Histological** Characteristics** (µm)**		**Treatments**					**L.S.D. 0.05**
		**Control**	**RL5**	**RL10**	**BL5**	**BL10**	
Upper epidermis thickness		24.89 ± 0.58^c^	29.40 ± 0.58^c^	49.39 ± 0.58^a^	37.94 ± 0.58^b^	34.43 ± 0.58^b^	1.49
Lower epidermis thickness		19.92 ± 0.58^d^	29.69 ± 0.58^c^	51.88 ± 0.58^a^	22.36 ± 0.58^d^	37.94 ± 0.58^b^	1.5
Lamina thickness		252.11 ± 0.89^e^	432.12 ± 0.59^d^	700.57 ± 0.89^a^	488.60 ± 0.89^c^	504.8 ± 0.89^b^	2.15
Palisade parenchyma thickness		83.40 ± 0.89^e^	102.39 ± 0.89^d^	184.13 ± 0.89^a^	146.16 ± 0.89^c^	174.92 ± 0.59^b^	2.15
Spongy parenchyma thickness		111.32 ± 0.59^d^	214.23 ± 0.89^c^	333.37 ± 1.21^a^	268.8 ± 0.89^b^	271.1 ± 0.88	2.35
Mesophyll tissue thickness		194.73 ± 0.89^e^	316.62 ± 1.16^d^	517.5 ± 0.88^a^	414.96 ± 1.17^c^	446.02 ± 0.58^b^	2.47
Midvein region thickness		423.7 ± 0.89^e^	605.9 ± 0.88^d^	742.4 ± 1.16^a^	635.90 ± 0.88^c^	735.7 ± 0.89^b^	2.43
Length	Dimension of the midvein bundle	206.0 ± 0.89^e^	235.5 ± 1.16^d^	271.1 ± 0.58^c^	288.7 ± 0.88^b^	328.3 ± 0.58^a^	2.17
Width		300.9 ± 0.89^c^	537.7 ± 0.58^a^	416.4 ± 0.89^d^	525.8 ± 1.16^b^	530.3 ± 0.89^a^	2.31
Xylem tissue thickness		72.4 ± 0.58^d^	113.4 ± 0.89^b^	83.0 ± 0.89^c^	118.9 ± 0.89^b^	143.6 ± 0.58^a^	2.007
Phloem tissue thickness		54.1 ± 0.88^c^	94.7 ± 0.84^a^	83.2 ± 0.58^b^	80.6 ± 0.89^b^	75.0 ± 0.89^b^	2.12
Xylem Vessel diameter		19.8 ± 0.59^c^	25.4 ± 0.84^b^	21.9 ± 0.59^b^	29.8 ± 0.89^b^	35.8 ± 0.89^a^	1.98

^*^Control: untreated plants, RL 5: plant treated with red laser for 5 min, RL 10: plant treated with red laser for 10 min, BL 5: plant treated with blue laser for 5 min, BL 10: plant treated with blue laser for 10 min

^*^The same superscript letters within a row designate there was no significant difference according to the Duncan multiple comparison test at (*p ≤* 0.05).* Each value is the mean of three replicates over one season. Means ± values indicated to standard error (± SE)

The cross-sectional data demonstrated that treated plants with red and blue laser irradiation for 5 and 10 min had a favorable effect on all histological parameters of sweet basil leaves when compared to untreated plants (Tables 8, Fig. 3). It was investigated that major anatomical parameters of leaves, such as the upper and lower epidermis, increased by 98.4 and 160.4% above the control, respectively after 10 min of red laser treatment.

Furthermore, this treatment resulted in thicker leaves than those of untreated plants. This extraordinary large rise can be due mostly to the significant thickness increase of both the midvein and lamina of sweet basil leaves, which are (75.2% and 177.8%) greater than those of the control plants, respectively.

In particular, exposure to red laser irradiation for ten min resulted in markedly thicker leaf blades, owing to increases in palisade (120.7%) and spongy tissues (199.4%). Furthermore, the mesophyll tissue, which is the specialized photosynthetic tissue containing chloroplasts in the palisade and spongy parenchyma, exhibited a 165.7% increase in thickness under the pre-sowing ten-minute red laser treatment compared with natural leaves. In addition, an increase of (31.6%) in length and (38.3%) in width was recorded in the midvein bundle in comparison with the control. Finally, the thickness of xylem and phloem tissues achieved higher and increased by 14.6 and 53.7% more than untreated plants as well as average vessel diameter increased by10.6% as compared to control.

The increase in palisade thickness may enhance photosynthetic capacity by improving light absorption, while the thicker cuticle and midvein structure could contribute to greater stress tolerance and water retention efficiency. Extremely high percentage values were verified and may result from tissue expansion, biological variation, or differences in section thickness during measurement.

Similarity, blue laser application for 10 min as shown in (Table 9, Fig. 4) recorded the highest significant increase in thickness of lamina of sweet basil leaves by 100.2% compared with the un-irradiated leaves (control). Such noticeable significant increase was mainly due to the marked thickening of both palisade (109.7%) and spongy tissues (143.5%), along with mesophyll tissue (129.0%), relative to untreated plants. Also, the increased percentage occurred by this treatment was 73.6% for midvein thickness over the control, respectively.

Furthermore, the primary vascular bundle of the midvein grew larger as a result of the same treatment. The dimensions of the midvein bundle rose by 59.3% in length and 76.2% in width, while xylem and phloem thickness increased by (98.3% and 38.6%) relative to the control, respectively. Also, the average diameter of vessels increased by (80.8%) in comparison with the control leaves.

Our findings are consistent with Metwally [37], who reported that umber of vascular bundles, bundle dimensions (length and width, μm), lamina thickness (μm), and midvein thickness (μm) showed significant increases after two- and three-minutes exposures to helium–neon laser rays in *Celosia argentea* plants. On the contrary, 6 min. exposure treatment recorded noticeable decrease for all most of previous mentioned parameters. Moreover, Zheng, and Van Labeke [58] demonstrated that 100% blue light and 75% red with 25% blue treatments increased the leaf thickness and resulted in a proportionate increase in palisade parenchyma which connected with leaf photosynthetic quantum efficiency in some ornamental plants.

On the other hand, Abou-Dahab [59] studied the effects of laser irradiation on the leaf anatomy of *Eustoma grandiflorum* plants. They found that 20 min of cadmium and 25 min of helium neon laser exposure resulted in the highest values of midvein thickness, lamina thickness, number of xylem rows, number of vessels, and vascular bundle dimensions (length and width) comparison with the control.

In the same line, Arafa [60] summarized that all He–Ne laser treatments increased the structural anatomy of Moringa leaves and recorded the highest values of lamina thickness, palisade tissue thickness, and spongy tissue thickness after being exposed to 15mw He–Ne laser power for 5 min as well as exposing seeds to 5 mW laser power for 5 min resulted in the highest value of thickness of the mid vein and mid vein bundle (length–width) as compared to control plants.

Hwida [38] reported that laser beams improved GA synthesis and encouraged IAA release, which had a positive impact on root growth, nutrient and water intake, as reflected in overall plant growth and leaf anatomy.

### Genetic diversity identification by SCoT, ISSR markers

The genetic variability of *O. basilicum* lines which treated with two laser exposure wavelengths (450 and 650 nm) for 5 and 10 min were examined using SCoT and ISSR analysis. The resulted bands exhibited the variation and similarities among the laser treated *Ocimum* lines and untreated plants.

**Fig. 5:**
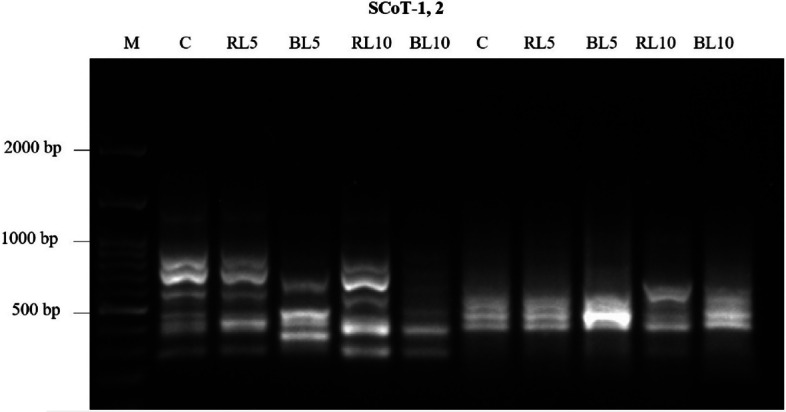
Banding patterns of four lines amplified with the ScoT “SCoT 1, SCoT 2, M:100 bp DNA ladder, Lanes C: Control untreated plants, Lanes RL5, RL10, BL5 and BL10: treated *Ocimum basilicum* plant lines. (C) untreated plants (control), (RL5) plant treated with red laser for 5 min, (RL10) plant treated with red laser for 10 min, (BL5) plant treated with blue laser for 5 min, (BL10) plant treated with blue laser for 10 min

According to the results of SCoT PCR products, a total number of 16 amplified fragments were recorded as shown in Table 10, Ten of these have a percentage polymorphism band of 62.5% and are polymorphic markers. With a different primer, a varied size of amplified fragments was obtained (280 to 1300 bp) (Fig. 5). the highest polymorphism percentage was 70% obtained by ScoT-1 with a total number of 10 bands. While SCoT-2 showed the lowest polymorphic pattern with a percentage of 50% (6 bands) (Table 10).

**Table 10:** Total band numbers, number of polymorphic bands and polymorphism percentages as revealed by SCoT, ISSR analyses

Primer** Name**	**Total No of bands**	**Polymorphic bands**	**Percentage of Polymorphism (%)**	**Band Size range (bp)**
SCoT-01	10	7	70	280- 1300
SCoT-02	6	3	50	290- 600
Total	16	10		
Average	8	10	60	
ISSR-03	12	3	25	160–910
ISSR-04	14	2	14	150- 1700
ISSR-05	9	2	22	190–770
ISSR-06	11	4	36	150- 870
ISSR-07	11	3	27	160- 950
ISSR-08	8	2	25	150- 515
ISSR-09	8	1	13	170- 570
ISSR-10	9	1	11	150- 560
Total	92	18		
Average	10.25	2.25	21.6

**Fig. 6:**
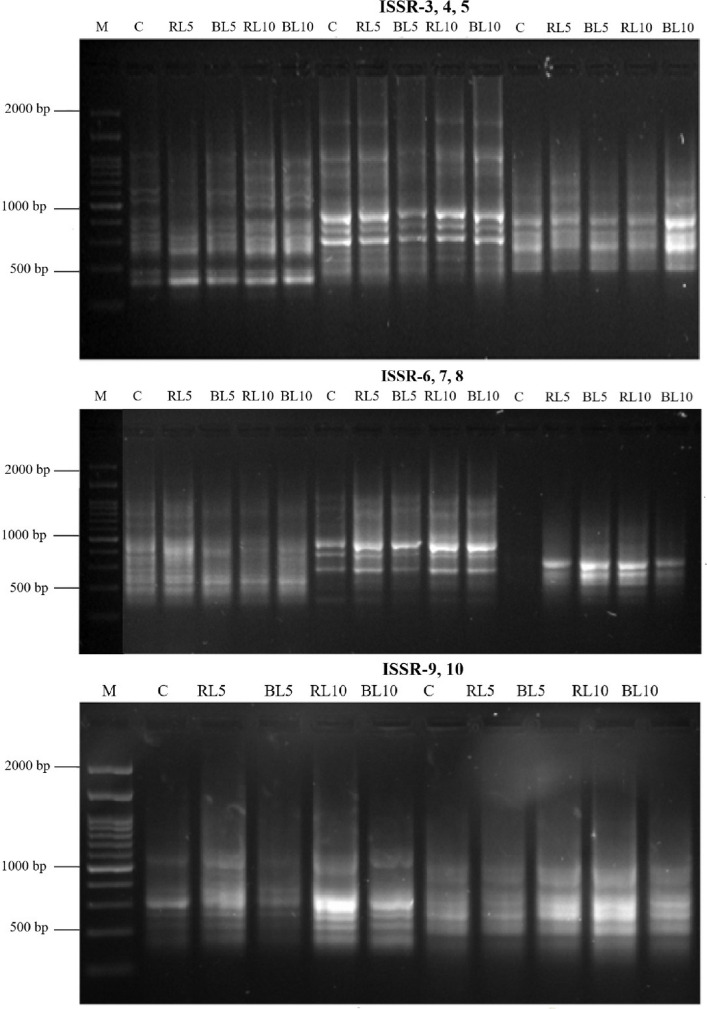
Banding patterns of ISSR primers “ISSR-3, ISSR-4, ISSR-5, ISSR-6, ISSR-7, ISSR-8, ISSR-9 and ISSR-10 M:100 bp DNA ladder, Lanes C: Control untreated plants, Lanes RL5, RL10, BL5 and BL10: treated *Ocimum basilicum* plant lines. (C) untreated plants (control), (RL5) plant treated with red laser for 5 min, (RL10) plant treated with red laser for 10 min, (BL5) plant treated with blue laser for 5 min, (BL10) plant treated with blue laser for 10 min

The genetic polymorphism between the control and irradiated* O. basilicum* lines was examined using eight ISSR primers that showed observable amplification profiles (Table 11, Fig. 6). The 8 ISSR primers produced 82 amplicons, out of which 18 were polymorphic with unique band, 15 were polymorphic without unique band and the average percentage of polymorphism was 21.6% (Table 11). Averaging 10.25 fragments/primer throughout the lines, the number of amplicons per primer varied from 8 (ISSR-8, ISSR-9) to 14 (ISSR-4).

**Table 11:** Genetic similarity coefficients based on SCoT, ISSR analysis among five Ocimum basilicum lines C, RL5 RL10, BL5, BL10

Treatments	C	RL5	RL10	BL5	BL10
C	100				
RL5	94.8	100			
RL10	89.8	89.3	100		
BL5	89.4	90.1	90.9	100	
BL10	92.2	91.7	91.4	93.3	100

^*^C: untreated plants (control), RL 5: plant treated with red laser for 5 min, RL 10: plant treated with red laser for 10 min, BL 5: plant treated with blue laser for 5 min, BL 10: plant treated with blue laser for 10 min

On the other hand, the average number of polymorphic amplicons was 2.25 fragments/primer, ranging from 1 (ISSR-9, ISSR-10) to 4 (ISSR-6). Two primers (ISSR-10 and ISSR-9) had low polymorphism (11 and 13%, respectively), whereas one primer (ISSR-6) displayed 36% polymorphism. Depending on the primer used, the amplified fragment's size ranged from 150 to 1700 bp (Fig. 6).

**Fig. 7:**
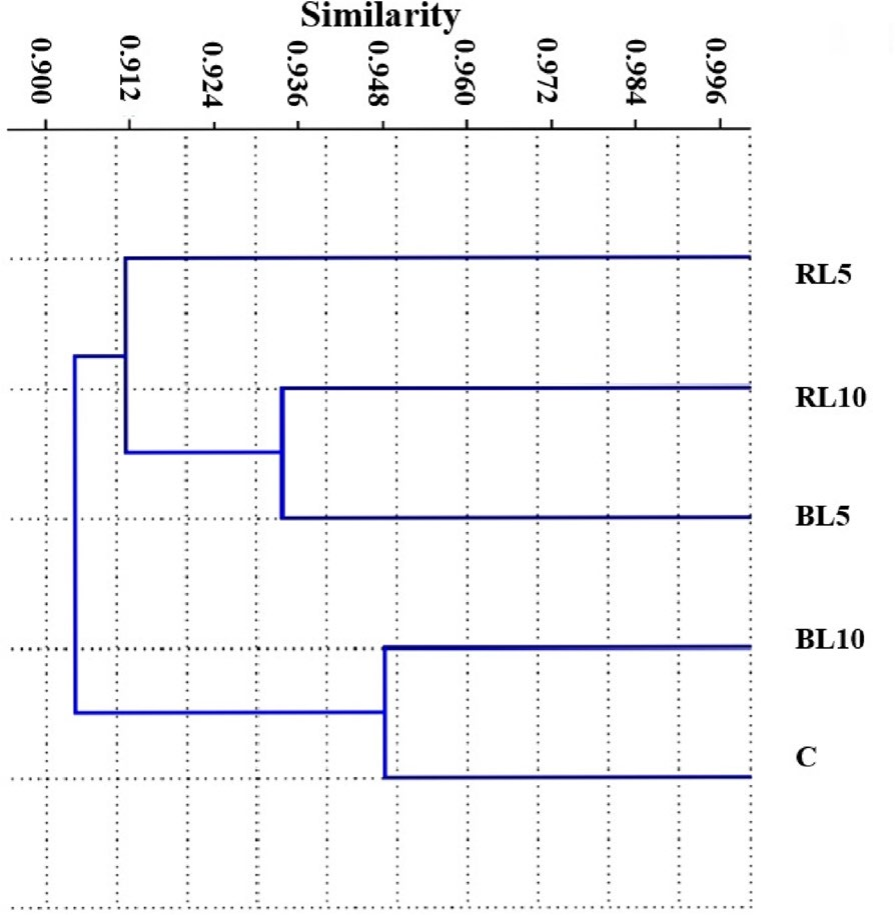
Dendrogram of five *Ocimum basilicum* lines cluster analysis using combined data of SCoT, ISSR markers. (C) untreated plants (control), (RL5) plant treated with red laser for 5 min, (RL10) plant treated with red laser for 10 min, (BL5) plant treated with blue laser for 5 min, (BL10) plant treated with blue laser for 10 min

Similarities were constructed using the scoring data from each marker type assay, and they were subsequently utilized in cluster analysis to produce dendrograms using UPGMA analysis to ascertain the genetic linkages among the five *O. basilicum* lines C, RL5 RL10, BL5, BL10 (Fig. 6). The data from both SCoT and ISSR analyses revealed that the first cluster was split into two sub-clusters consists of the RL5 and control plants. The second group was split into two sub-clusters first one consists of BL5 and BL10 line. The second sub cluster one contained RL10 plant line (Fig. 7).

The percentage of polymorphism among the laser-treated and control basil plants which detected by SCOT and ISSR markers reflects that higher polymorphism percentages suggest that laser exposure may have altered the genomic template at certain loci or affected the regulation of gene regions targeted by these markers. Because SCOT markers are gene-targeted, polymorphic changes in SCOT profiles can imply modulation of gene expression or structural variation near coding regions. In contrast, ISSR markers amplify regions between microsatellite repeats that are often noncoding, and increased polymorphism there may indicate random DNA alterations, replication errors, or DNA repair events triggered by oxidative or photophysical stress induced by laser irradiation.

From the obtained results of both SCoT and ISSR tests, the genetic similarity of the five *Ocimum* lines—1: RL5, 2: RL10, 3: BL5, 4: BL10, and C: Control plants—was ascertained. According to the results, RL5 and C had the closest resemblance (0.95), whereas RL5 and RL10 Ocimum lines had the lowest similarity (0.89). The first cluster is separated into two sub-clusters (C and RL5), while the second group is likewise divided into two sub-clusters (BL5 and BL10), according to a cluster analysis of the combined data from SCoT and ISSR analyses. However, only the RL10 *Ocimum* line was present in the second group.

Table 10 shows that there were 16 SCoT amplicons overall, with an average of 8 amplicons/primer, and that the product size ranged from 280 to 1300 bp when comparing the data for both SCoT and ISSR primers. The product size ranged from 150 to 1700 bp, and there were 82 total scoreable ISSR amplicons, with an average of 10 amplicons/primer.

Additionally, in comparison to SCoT and ISSR primers, 10 polymorphic amplicons with a 60% percentile were produced. While ISSR primers targeted generating 18 polymorphic amplicons with a percent of 21.6% polymorphism.

These results agree previously by Ahmed [61] who showed that the highest number of species-specific markers was generated by SCoT analysis as compared to the ISSR analysis. This also confirmed by Abd El-Hadi [62] who considered both ISSR and SCoT PCR as a useful marker for distinguishing between four basil varieties. Overall, the moderate-to-high polymorphism percentages observed in laser-treated basil plants indicate that the laser light affecting the genomic profile and could enhancing genetic variability.

High polymorphism of SCoT was also reported previously in *Ocimum* plants by Gupta [63]. Furthermore, in *Ocimum* plants, ISSR markers perform better than alternative molecular marker systems [63, 64]. In contrast to the SCoT marker, the ISSR marker system is helpful for identifying *Ocimum* species and analyzing their genetic diversity. The current study and a number of earlier genetic diversity studies that examined species of the genus *Ocimum* proved the effectiveness of the ISSR [61].

Overall, the appearance of polymorphic and unique bands by SCoT and ISSR markers constitutes definite proof that laser exposure caused detectable genomic changes in the treated *O. basilicum* lines. In the case of the SCoT markers, the relatively high percentage of polymorphism is related to the sensitivity of gene-rich regions, especially those around the start codon, to stress-induced modifications. The emergence of new bands after treatment indicated that some loci had been activated or structurally changed; disappearance of bands, on the other hand, probably showed sequence changes or methylation events which prevented primer binding. Although ISSR markers yielded a lower overall polymorphism, the presence of several unique bands within treated lines proved microsatellite-associated regions were indeed affected-although to a much lesser extent compared with SCoT-targeted sites.

The clustering pattern supports these molecular observations. The close grouping of the A1 line with the control, together with their high similarity coefficient, 0.95, indicates that this treatment caused only minor genomic variation. By contrast, the distinct placement of A2 in a separate sub-cluster, accompanied by the lowest similarity values among all lines, reflects more substantial genetic divergence consistent with its higher band polymorphism. Similarly, the tight clustering of B1 and B2 suggests that their respective treatments induced comparable genomic responses. Therefore, the distances separating clusters in the dendrogram represent the cumulative impact of band gains, losses, and polymorphisms across marker systems, with greater genetic distance corresponding to more pronounced molecular changes. Taken together, these patterns confirm that different wavelengths and exposure durations elicit distinct levels of genomic stress, measurable through both SCoT and ISSR marker profiles.

## Conclusion

The present study evaluated the impact of red and blue laser irradiation in different exposure time on sweet basil (*Ocimum basilicum* L.) seeds. Sweet basil seeds treated with red and blue laser rays for 10 min induce a range of positive changes in plants against the un-irradiated seeds for most of the parameters. These changes involve morphological, biochemical, physiological, anatomical and genetic molecular adjustments, ultimately leading to improved vegetative growth, stimulated the biosynthesis of GA3 and IAA hormones. In addition, it enhanced the photosynthetic pigments in the treated plant leaves such as (chlorophyll a, b and total carotenoids). Also, it promoted the synthesis of protective metabolites (phenolic compounds) and increased the antioxidant enzymes' activities (CAT and GPX). Furthermore, the same treatments cause extraordinary increase in the leaf anatomical structure of basil plants over the control. The genetic variability analysis showed that the SCoT and ISSR marker systems revealed distinct levels of polymorphism among *Ocimum basilicum* plants exposed to laser irradiation. The present findings validate that laser irradiation can serve as a reliable, eco-friendly bio-stimulation tool to improve basil growth, biochemical composition, and genetic performance without chemical inputs. The results can be practically applied in sustainable agriculture programs to enhance crop productivity and phytochemical yield while minimizing environmental impact. Future studies should focus on optimizing laser parameters and integrating this technology with precision agriculture systems to strengthen its application in large-scale crop improvement.

## Supplementary Information

Supplementary Material 1.

Supplementary Material 2.

Supplementary Material 3.

Supplementary Material 4.

Supplementary Material 5.

Supplementary Material 6.

Supplementary Material 7.

## Data Availability

The datasets generated for this study are available in the published manuscript.
