# Supplementary material for: Effects of laser irradiation on phytochemical composition, histological anatomy, genetic diversity, and food safety of Ocimum basilicum L
Source: BMC Plant Biol. 2026 Feb 9;26:381. doi: 10.1186/s12870-026-08136-2 (PMC12931005; doi:10.1186/s12870-026-08136-2)
Supplement: Supplementary file 1 — Supplementary Material 1. [file 12870_2026_8136_MOESM1_ESM.pdf]

Image Report: 1 and 2

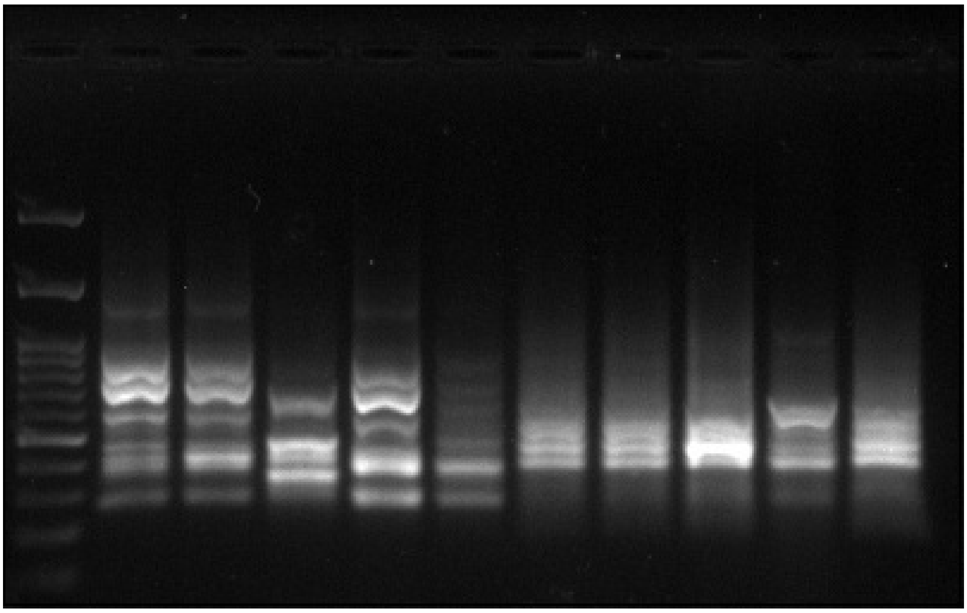

D:\Dr amal\1 and 2.scn

Acquisition Information

Imager	Gel Doc™ XR+
Exposure Time (sec)	0.666 (Auto - Intense Bands)
Dark Type	Referenced
Ref. Bkgd. Time (sec)	20
Serial Number	721BR12482
Software Version	5.2.1
Application	Ethidium Bromide
Excitation Source	UV Trans illumination
Emission Filter	Standard Filter

Image Information

Acquisition Date	9/23/2024 1:51:58 PM
User Name	Dell
Image Area (mm)	X: 75.3 Y: 47.3
Pixel Size (um)	X: 186.8 Y: 186.8
Data Range (Int)	142 - 3970

Analysis Settings

No analysis performed
