# Supplementary material for: Effects of laser irradiation on phytochemical composition, histological anatomy, genetic diversity, and food safety of Ocimum basilicum L
Source: BMC Plant Biol. 2026 Feb 9;26:381. doi: 10.1186/s12870-026-08136-2 (PMC12931005; doi:10.1186/s12870-026-08136-2)
Supplement: Supplementary file 5 — Supplementary Material 5. [file 12870_2026_8136_MOESM5_ESM.docx]

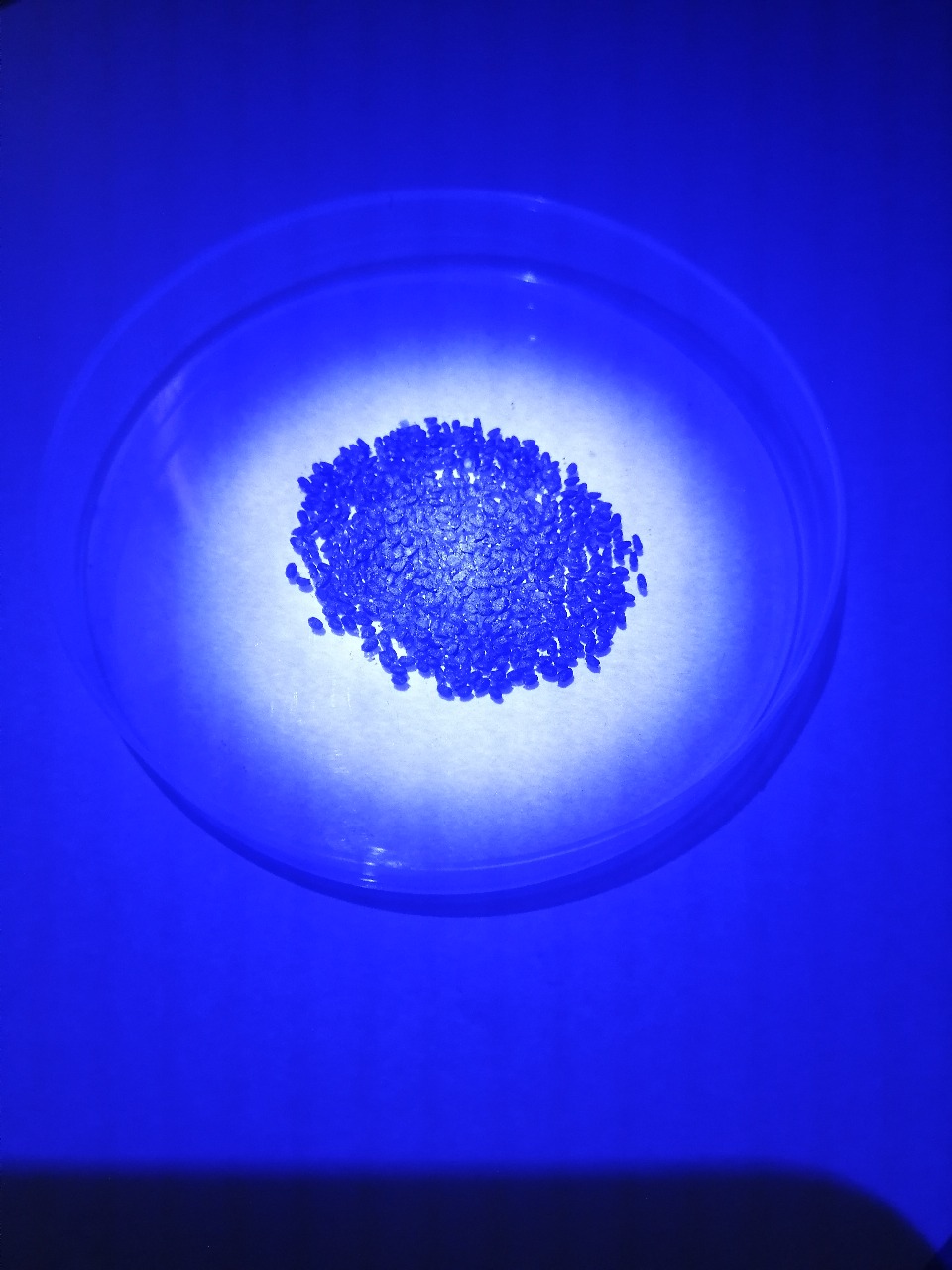


**Fig.1a: Blue Laser**


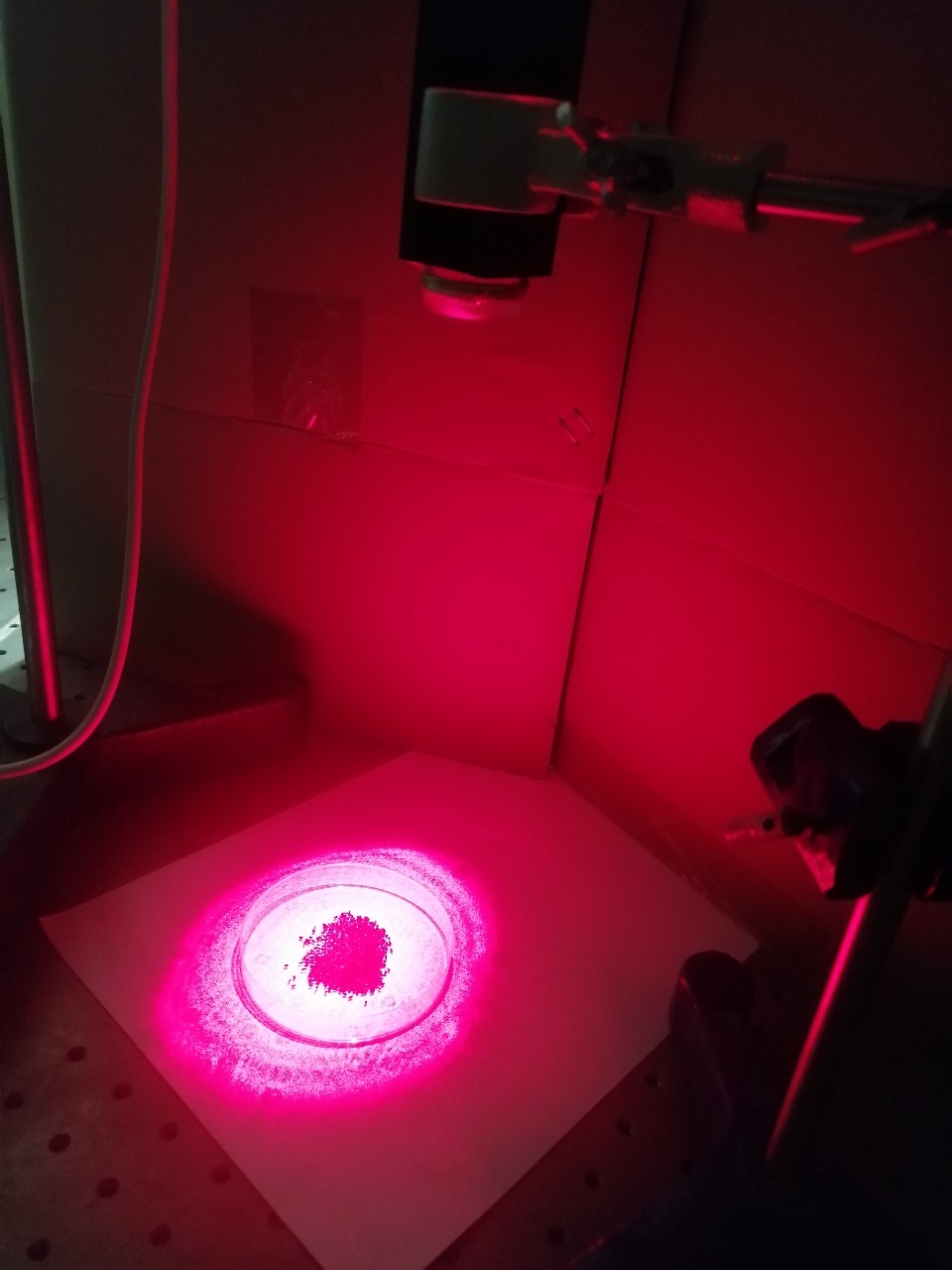


**Fig.1b: Red Laser**

**Figure 1.** Irradiation of sweet basil seeds by (a) blue laser 450nm, ,100mW, (b) red laser 650nm,100mW


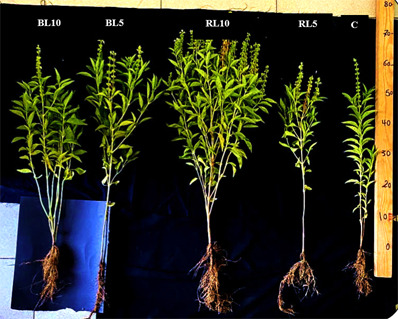


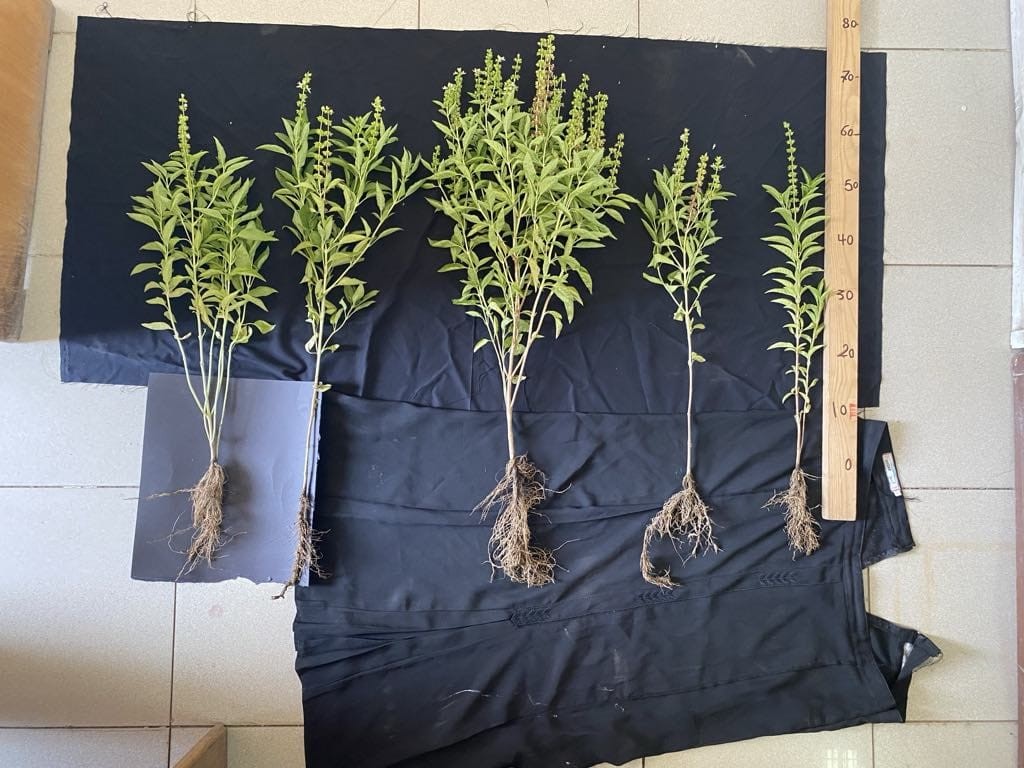


RL10

Fig.2

BL10

BL5

C

RL5

**Figure 2.** Comparative effects of laser irradiation at different exposure times on phenotypic changes of sweet basil plants, (C) untreated plants (control), (RL5) plant treated with red laser for 5 min, (RL10) plant treated with red laser for 10 min, (BL5) plant treated with blue laser for 5 min, (BL10) plant treated with blue laser for 10 min.


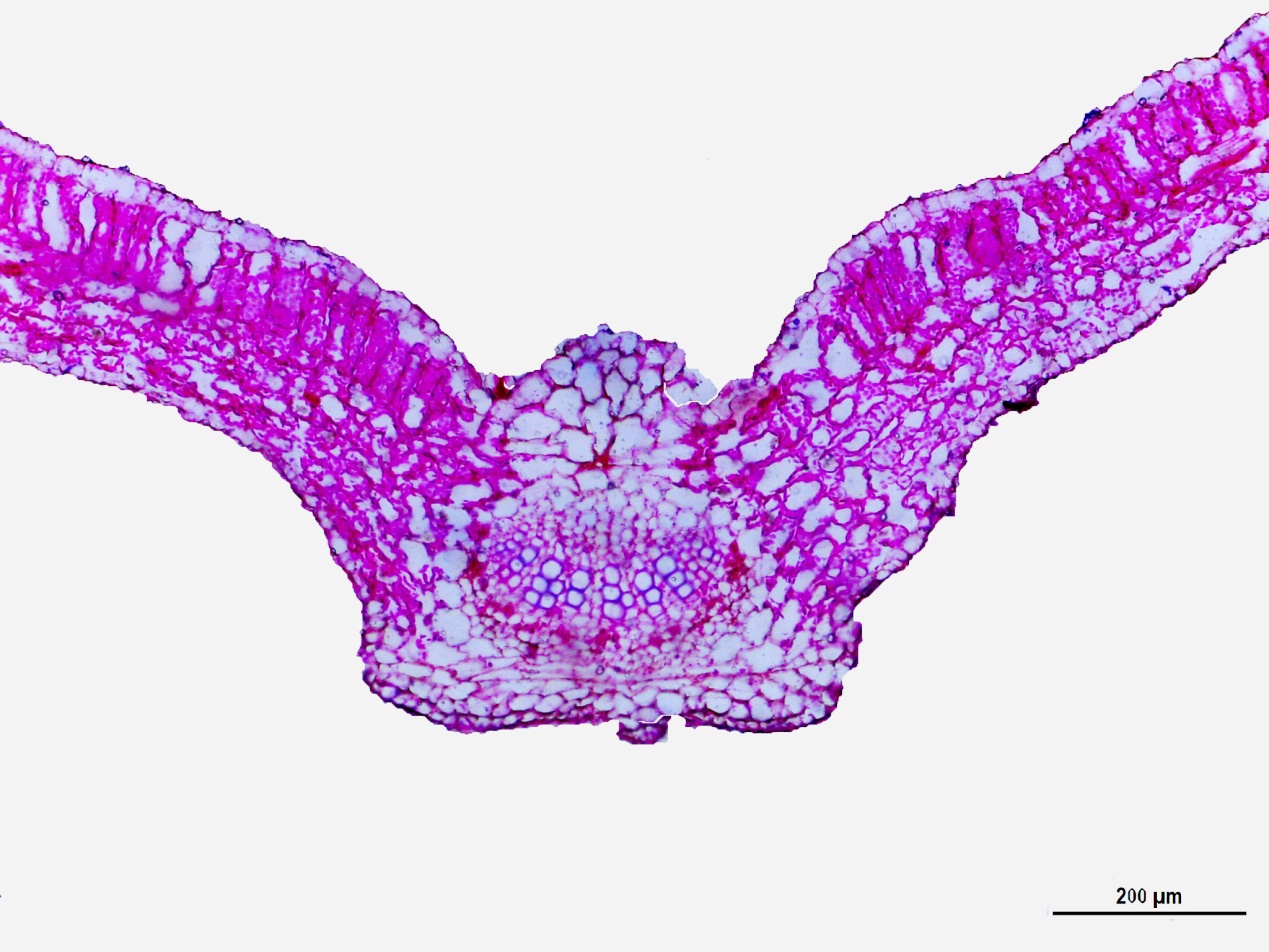


**Fig3.C**

mid r

xy

sp

pa

l ep

u ep


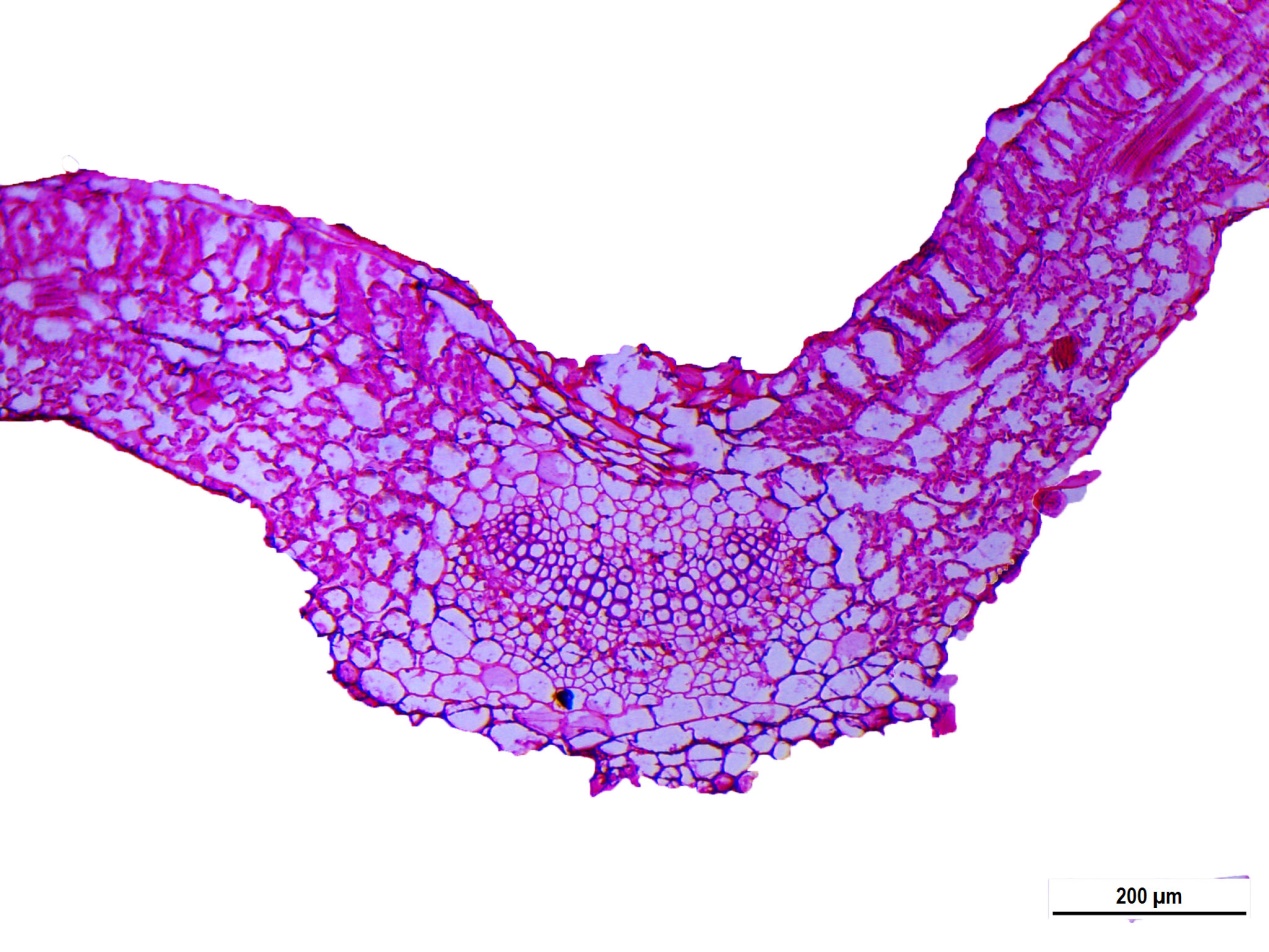


**Fig3.RL5**

xy

ph


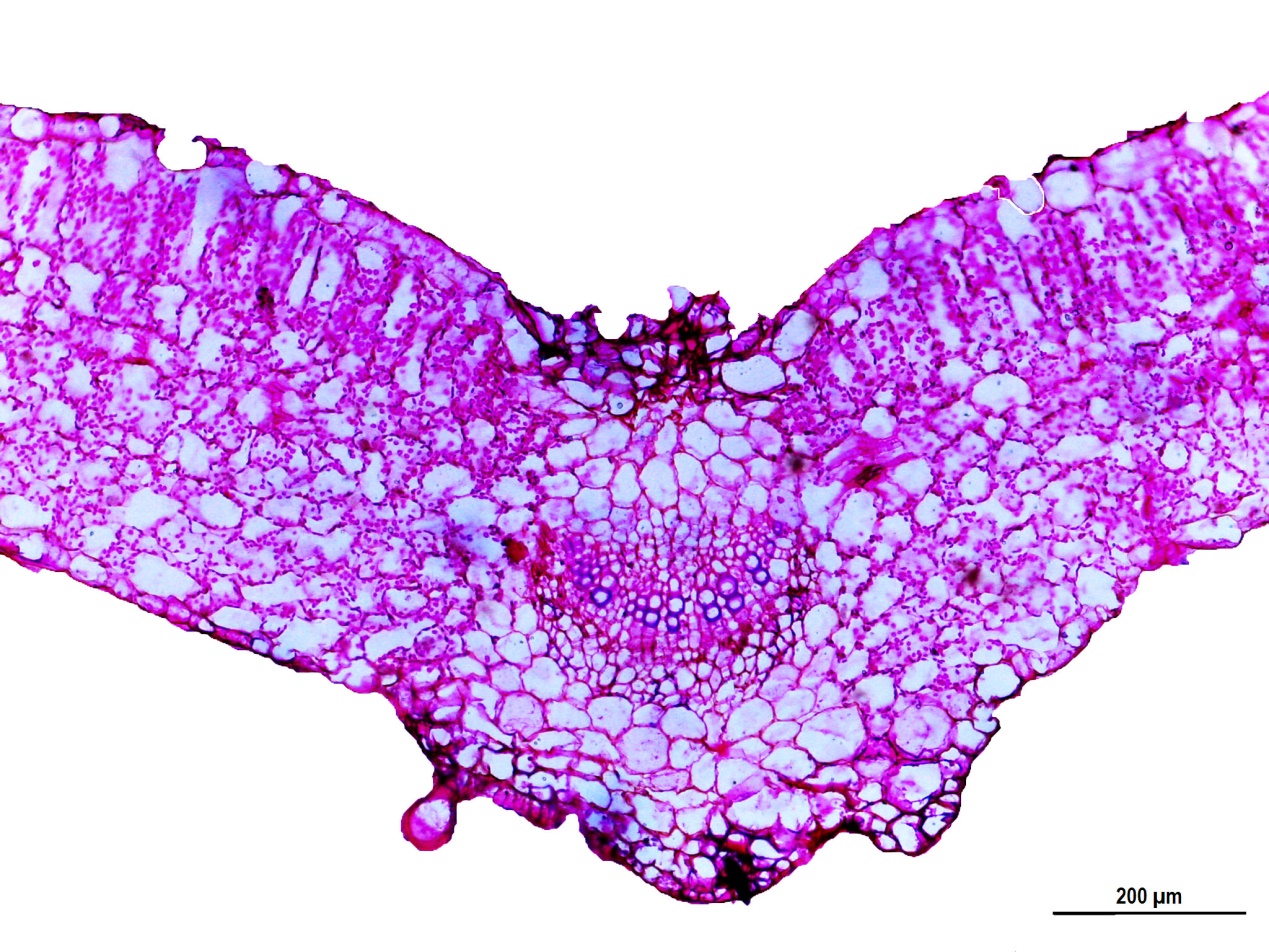


**Fig3.RL10**

pa

u ep

sp

l ep


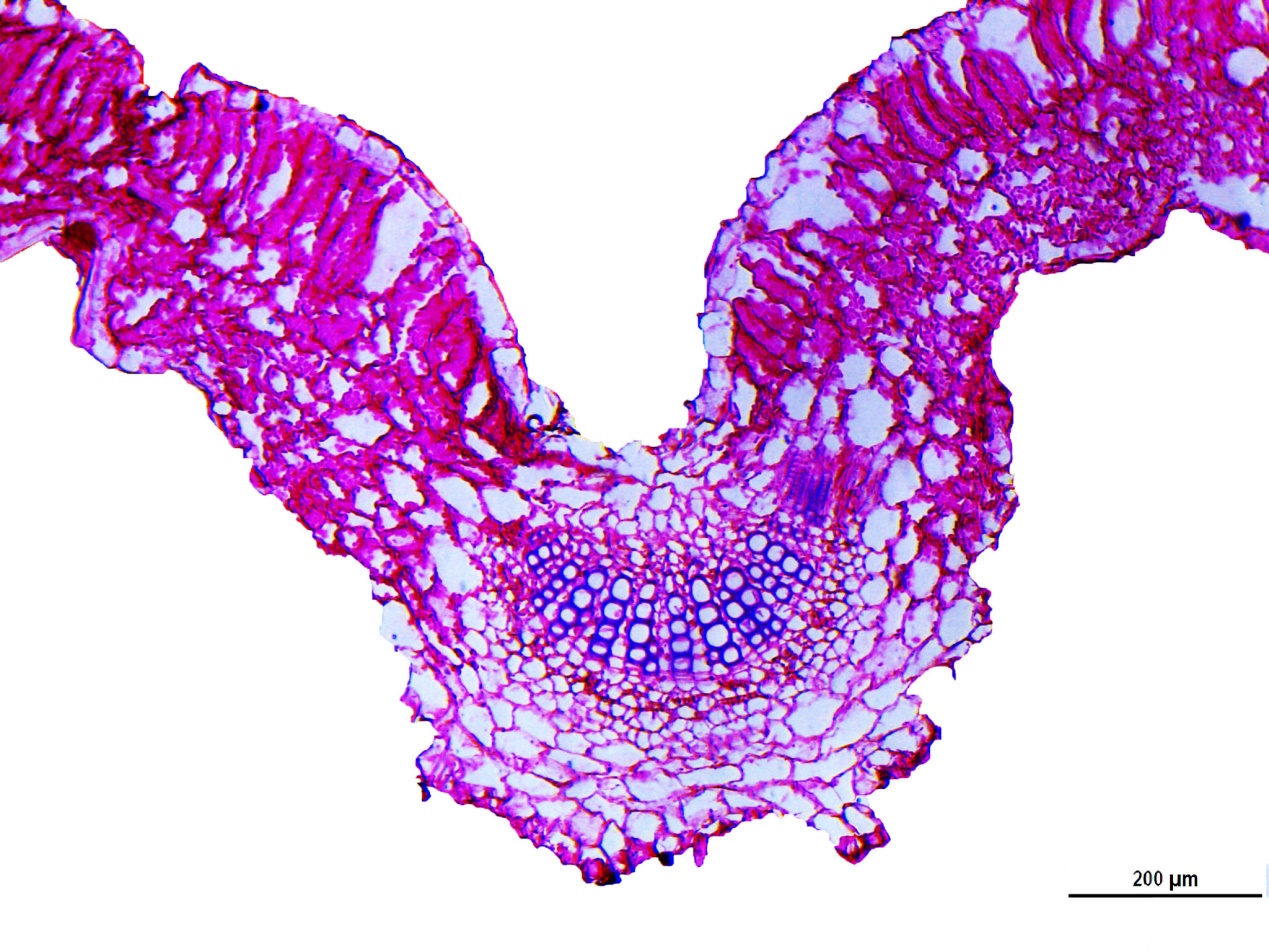


**Fig3.BL5**

mid b

ph

xy


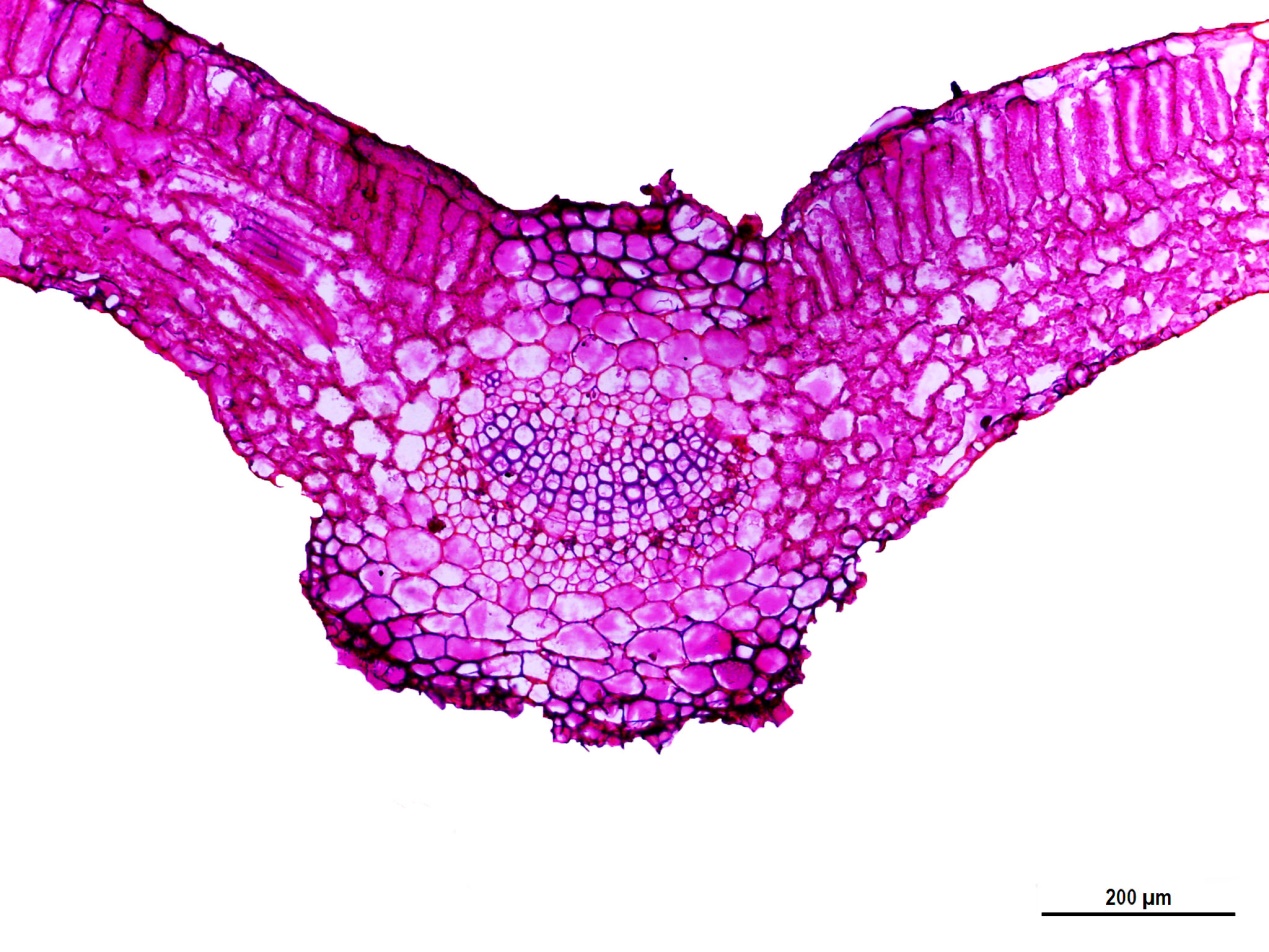


**Fig3.BL10**

sp

pa

ph

mid r

xy


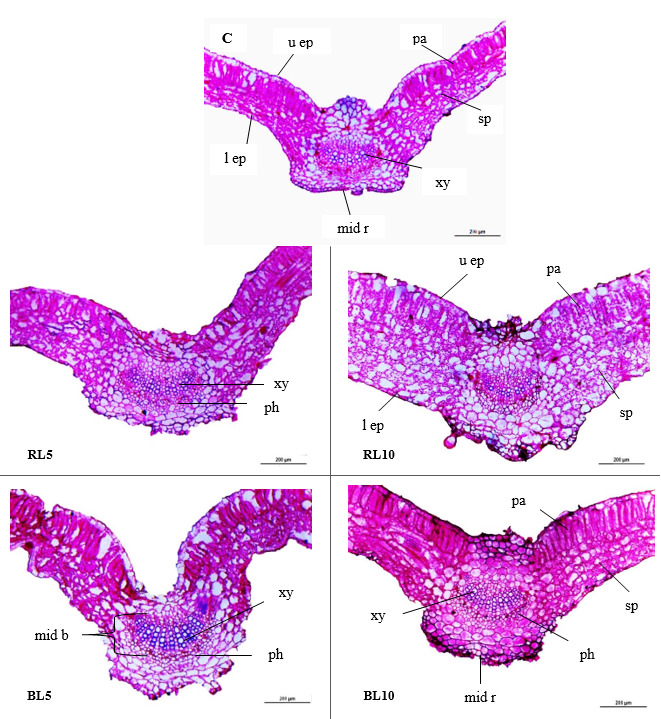


**Figure 3.** Transverse sections through the leaf blade of the fourth node developed on the median portion of the main stem of sweet basil plants as treatments by RL5, RL10, BL5, BL10 and Control in 2024 (first) season (200x). Where: up ep= upper epidermis, l ep= lower epidermis, pa= palisade parenchyma, sp= spongy parenchyma, mid b= midvein bundle, mid r= midrib region, xy= xylem tissue, ph= phloem tissue.


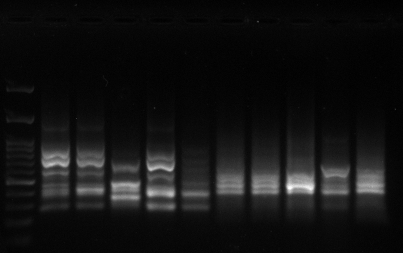


Fig. 4: SCoT 1 ,2


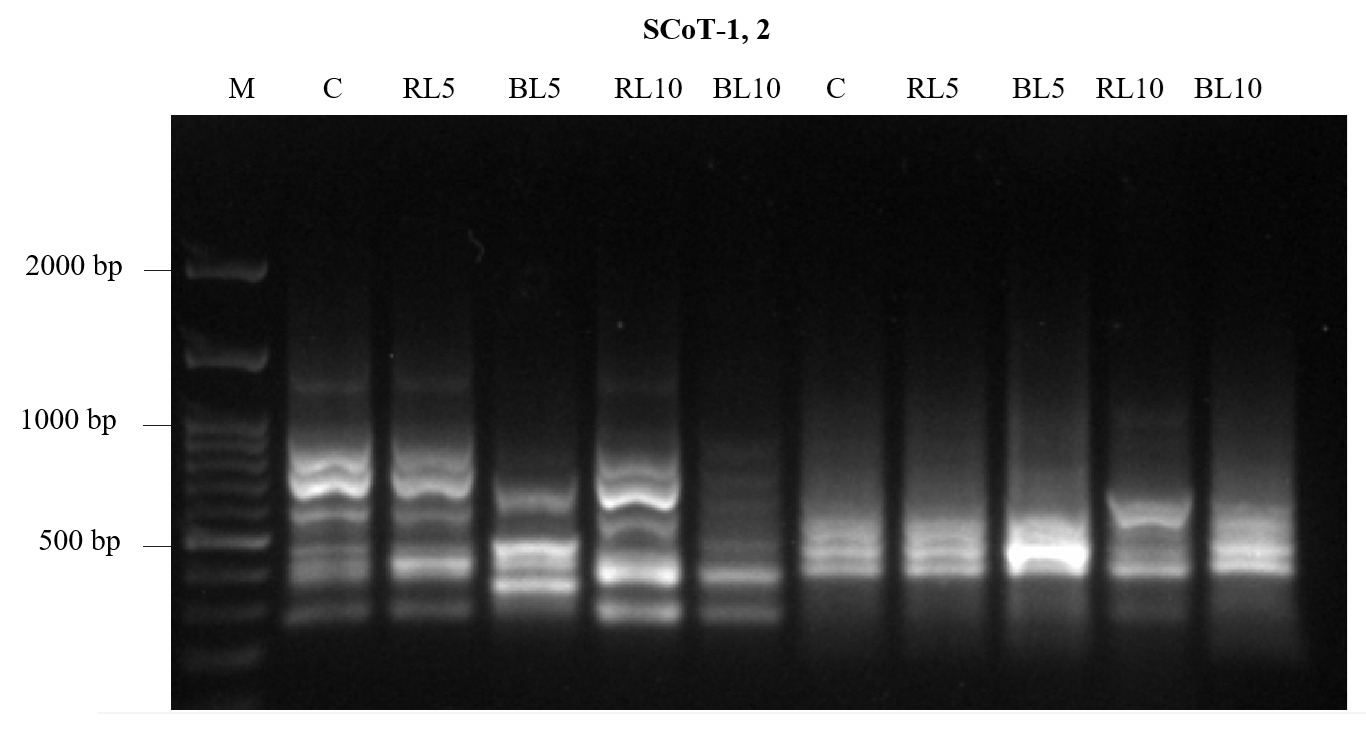


**Figure 4.** Banding patterns of four lines amplified with the ScoT “SCoT 1, SCoT 2**”** **M: 1000bp DNA ladder, Lane C: Control untreated plants, Lanes A1, A2, B1 and B2: treated *Ocimum basilicum* plant lines..**

**
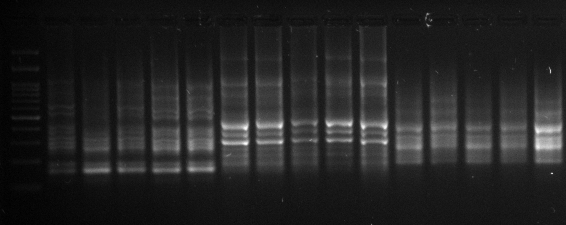
**

Fig. 5: SCoT3,4,5

**Figure 5. Banding patterns of four lines amplified with the ISSR primers “ISSR-03, ISSR-04, ISSR-05. M: 1000bp DNA ladder, Lane C: Control untreated plants, Lanes A1, A2, B1 and B2: treated *Ocimum basilicum* plant lines.**


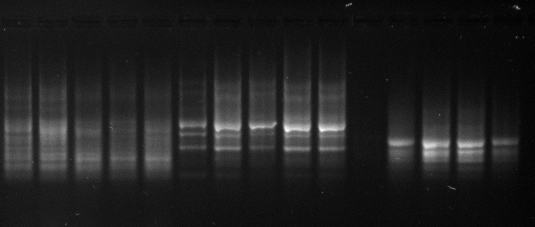


Fig. 5: SCoT6,7,8

**Figure 5. Banding patterns of four lines amplified with the ISSR-06, ISSR-07, ISSR-08. M: 1000bp DNA ladder, Lane C: Control untreated plants, Lanes A1, A2, B1 and B2: treated *Ocimum basilicum* plant lines.**

**
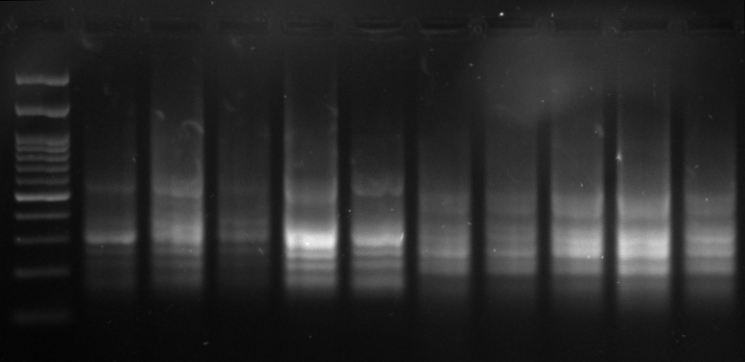
**

Fig. 5: SCoT9,10

**Figure 5. Banding patterns of four lines amplified with the ISSR-09 and ISSR-10 M: 1000bp DNA ladder, Lane C: Control untreated plants, Lanes A1, A2, B1 and B2: treated *Ocimum basilicum* plant lines.**


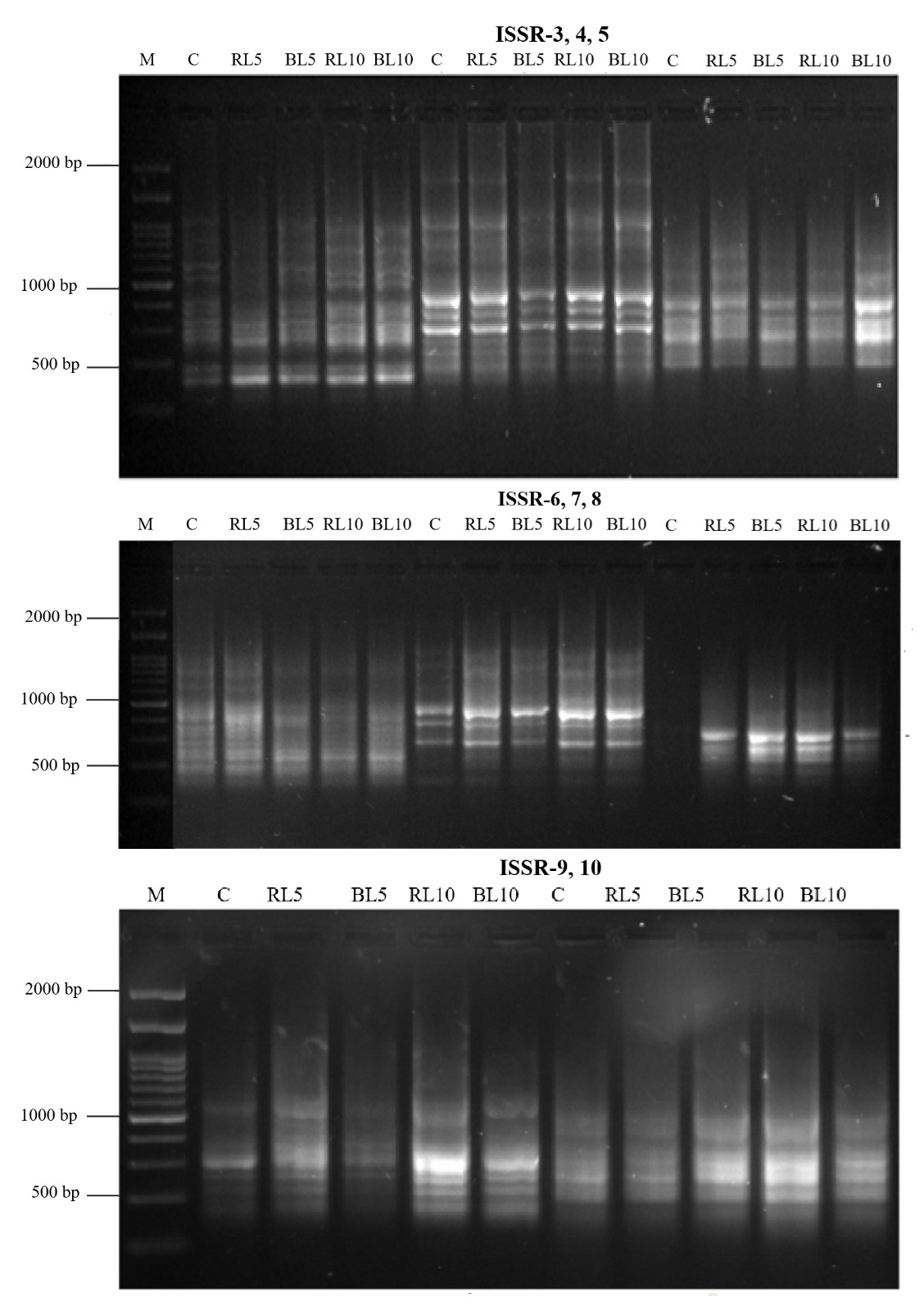


**Figure 5.** Banding patterns of ISSR primers “ISSR-3, ISSR-4, ISSR-5, ISSR-6, ISSR-7, ISSR-8, ISSR-9 and ISSR-10 M:100bp DNA ladder, Lanes “C, RL5, RL10, BL5 and BL10.

**
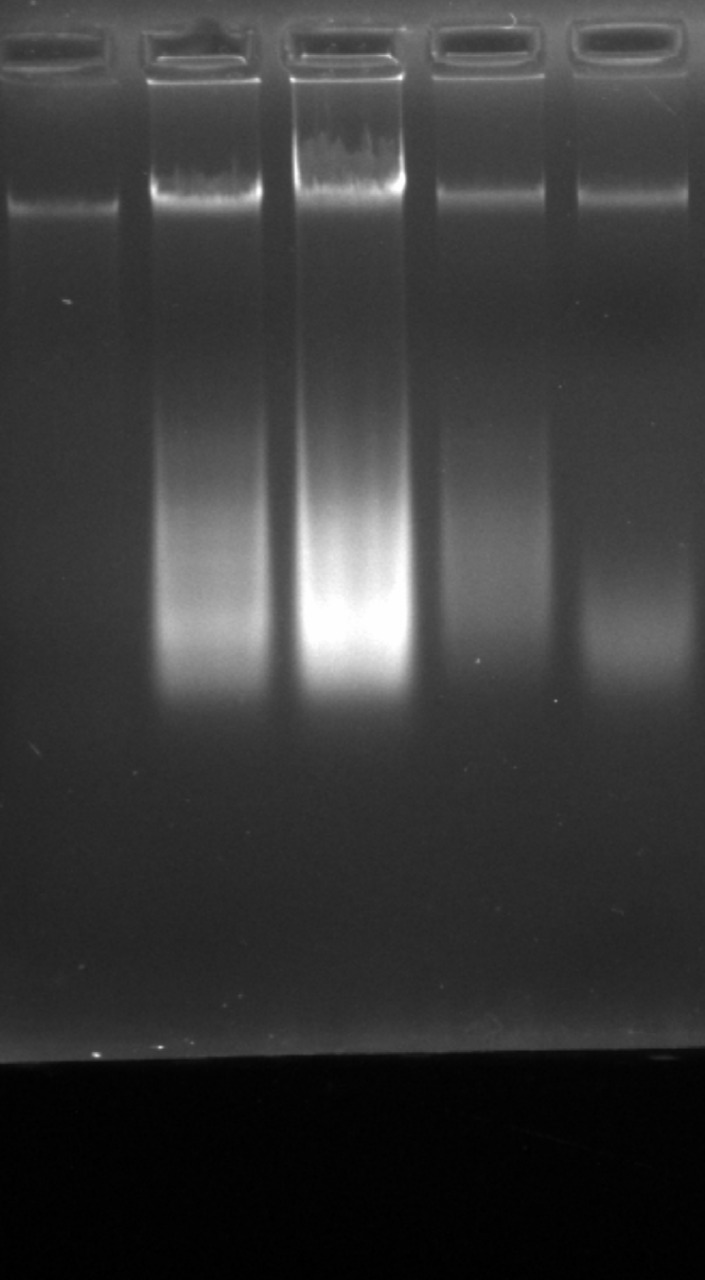
**

**Fig.: DNA extraction from five *Ocimum basilicum* plant lines; Lane 1: Control untreated plant Lane 2: A1 treated plant line. Lane 3: A2 treated plant line. Lane 4: B1 treated plant line.**


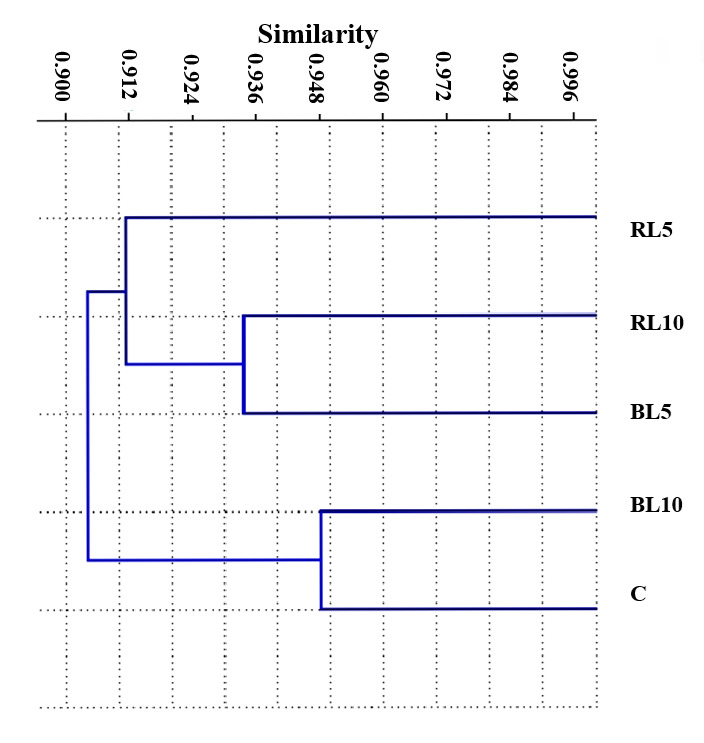


**Figure 6.** Dendrogram of five *Ocimum basilicum* lines cluster analysis using combined data of SCoT, ISSR markers.
