# Supplementary figures and images for: Effects of laser irradiation on phytochemical composition, histological anatomy, genetic diversity, and food safety of Ocimum basilicum L
Source: BMC Plant Biol. 2026 Feb 9;26:381. doi: 10.1186/s12870-026-08136-2 (PMC12931005; doi:10.1186/s12870-026-08136-2)

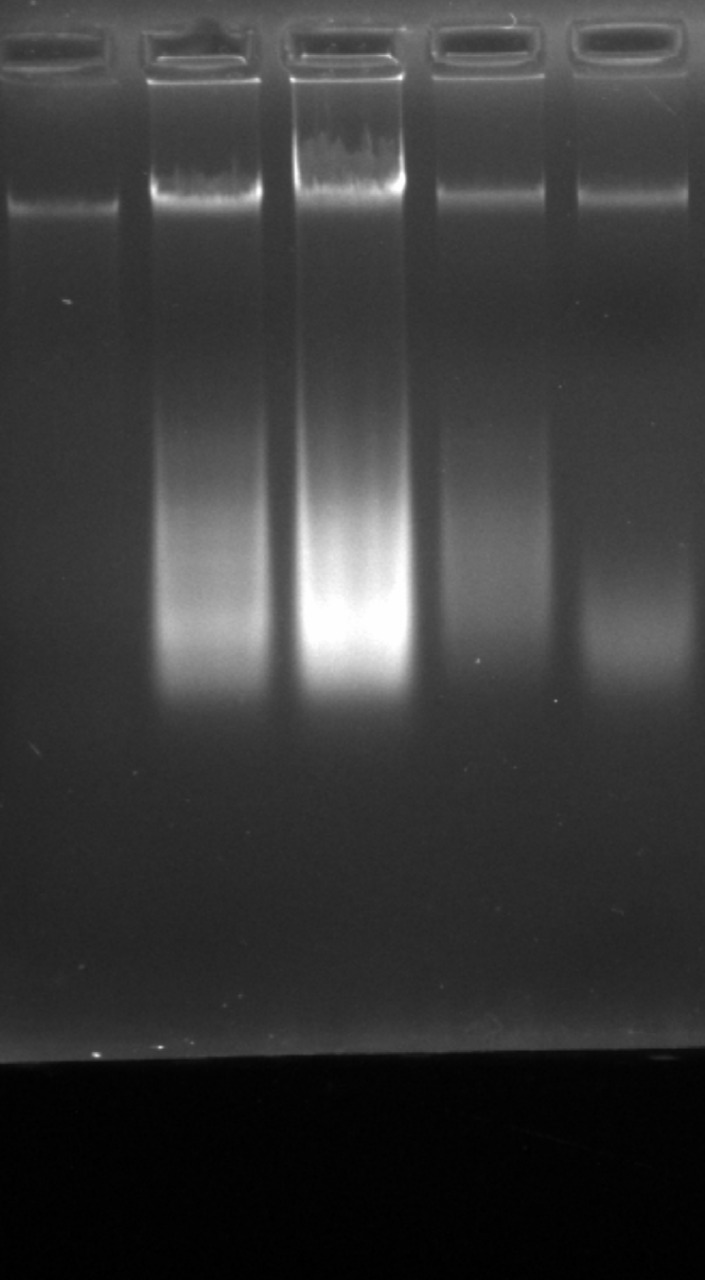

Supplement: Supplementary file 6 — Supplementary Material 6. [file 12870_2026_8136_MOESM6_ESM.jpeg]
