# Supplementary material for: Effects of laser irradiation on phytochemical composition, histological anatomy, genetic diversity, and food safety of Ocimum basilicum L
Source: BMC Plant Biol. 2026 Feb 9;26:381. doi: 10.1186/s12870-026-08136-2 (PMC12931005; doi:10.1186/s12870-026-08136-2)
Supplement: Supplementary file 7 — Supplementary Material 7. [file 12870_2026_8136_MOESM7_ESM.pdf]

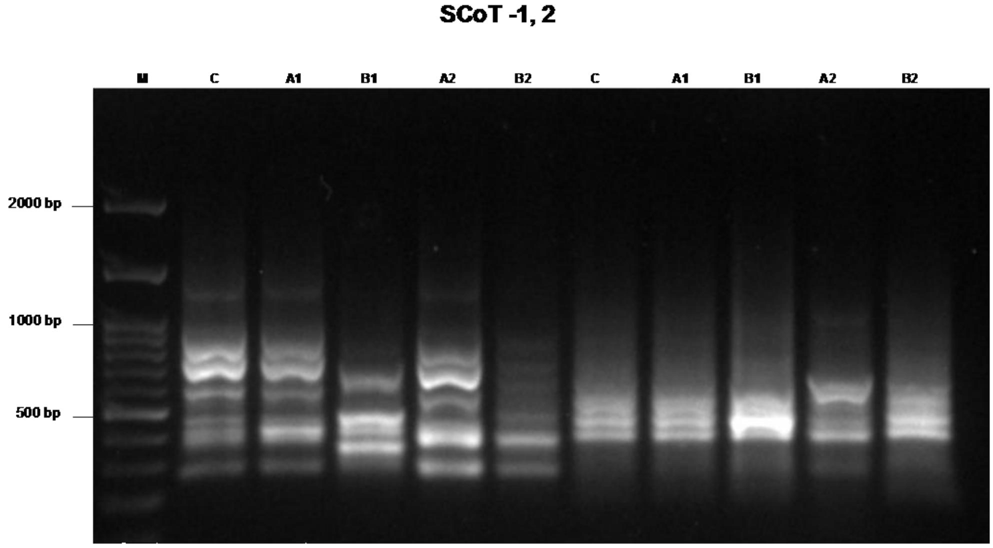

Fig.: Banding patterns of four lines amplified with the Scot 1 and Scot 2.

M: 1000bp DNA ladder, Lane C: Control untreated plants,

Lanes A1, A2, B1 and B2: treated *Ocimum basilicum* **plant** lines.

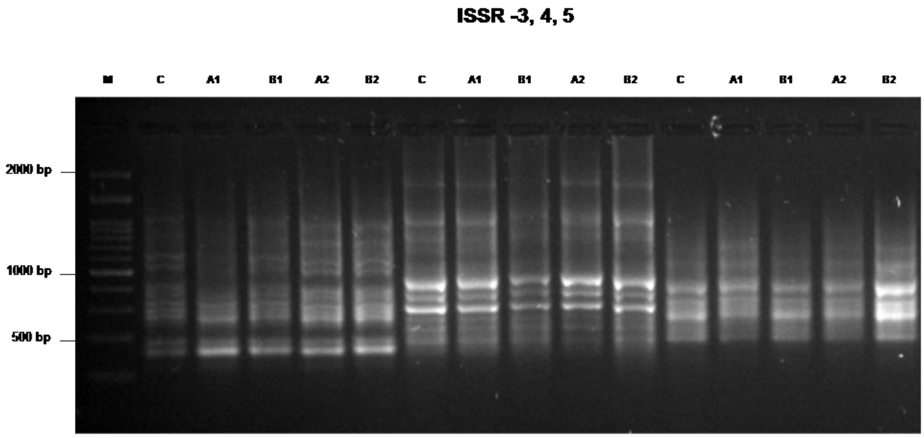

Fig.: Banding patterns of four lines amplified with the ISSR-03, ISSR-04 and ISSR-05.

M: 1000bp DNA ladder, Lane C: Control untreated plants,

Lanes A1, A2, B1 and B2: treated *Ocimum basilicum* **plant** lines.

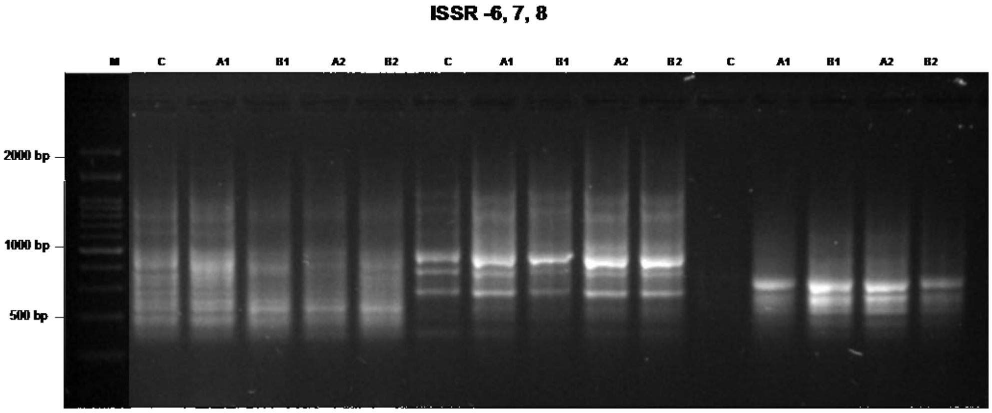

Fig.: Banding patterns of four lines amplified with the ISSR-06, ISSR-07 and ISSR-08.

M: 1000bp DNA ladder, Lane C: Control untreated plants,

Lanes A1, A2, B1 and B2: treated *Ocimum basilicum* **plant** lines.

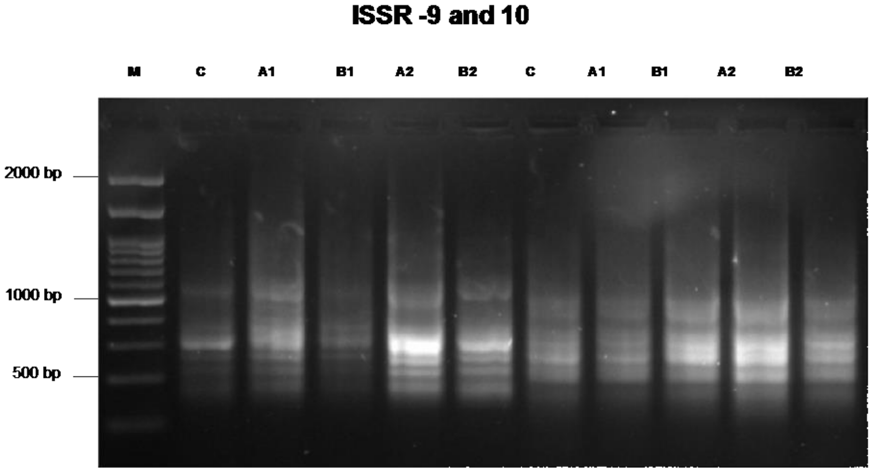

Fig.: Banding patterns of four lines amplified with the ISSR-09 and ISSR-10.

M: 1000bp DNA ladder, Lane C: Control untreated plants,

Lanes A1, A2, B1 and B2: treated *Ocimum basilicum* **plant** lines.

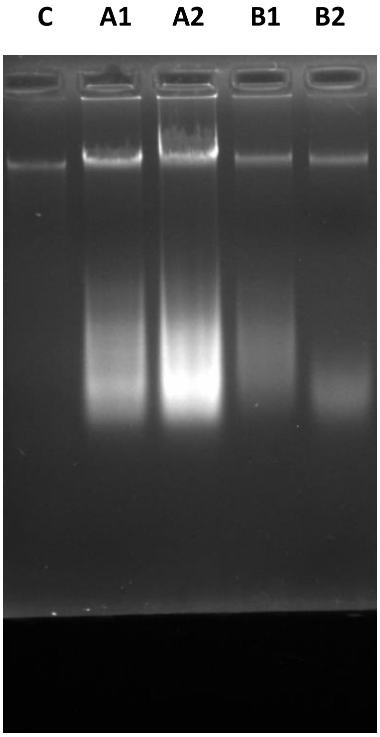

Fig.: DNA extraction from five *Ocimum basilicum* plant lines;

Lane 1: Control untreated plant

Lane 2: A1 treated plant line.

Lane 3: A2 treated plant line.

Lane 4: B1 treated plant line.

Lane 5: B2 treated plant line.
